# Histone Modification Landscapes as a Roadmap for Malaria Parasite Development

**DOI:** 10.3389/fcell.2022.848797

**Published:** 2022-04-01

**Authors:** J. Connacher, H. von Grüning, L. Birkholtz

**Affiliations:** Malaria Parasite Molecular Laboratory, Department of Biochemistry, Genetics and Microbiology, Institute for Sustainable Malaria Control, University of Pretoria, Pretoria, South Africa

**Keywords:** histone post-translational modifications, histone combinations, epigenetic regulation, malaria, *Plasmodium*, gametocyte

## Abstract

*Plasmodium falciparum* remains the deadliest parasite species in the world, responsible for 229 million cases of human malaria in 2019. The ability of the *P. falciparum* parasite to progress through multiple life cycle stages and thrive in diverse host and vector species hinges on sophisticated mechanisms of epigenetic regulation of gene expression. Emerging evidence indicates such epigenetic control exists in concentric layers, revolving around core histone post-translational modification (PTM) landscapes. Here, we provide a necessary update of recent epigenome research in malaria parasites, focusing specifically on the ability of dynamic histone PTM landscapes to orchestrate the divergent development and differentiation pathways in *P. falciparum* parasites. In addition to individual histone PTMs, we discuss recent findings that imply functional importance for combinatorial PTMs in *P. falciparum* parasites, representing an operational histone code*.* Finally, this review highlights the remaining gaps and provides strategies to address these to obtain a more thorough understanding of the histone modification landscapes that are at the center of epigenetic regulation in human malaria parasites.

## Introduction

Malaria persists as a global burden to public health and in 2019, was responsible for 409000 deaths. Of the five *Plasmodium* species that infect humans, *P. falciparum* is the most likely to cause severe disease and accounts for the vast majority of deaths from malaria (WHO, 2020). The complexities of the *P. falciparum* life cycle are evident from the array of developmental stages and cycles that are compartmentalized into diverse host cell types within both the human and the mosquito vector (Sherman, 1979). The unique biology of each stage is underpinned by the expression of stage-specific gene sets, regulated at the epigenetic, transcriptional, post-transcriptional, and post-translational levels (Bozdech et al., 2003; Young et al., 2005; Lopez-Barragan et al., 2011; van Biljon et al., 2019). Recent studies involving a diverse set of chromatin-based technologies, including a combination of chromatin immunoprecipitation coupled with next-generation sequencing (ChIP-seq), quantitative mass spectrometry (MS), fluorescence *in situ* hybridization (FISH), and histone mutagenesis and transcriptomics (microarrays and RNA-sequencing), have revealed that histone post-translational modifications (PTMs) are foundational in generating these stage-specific gene expression fingerprints and are thus proposed to be central to *P. falciparum* parasite life cycle regulation (Comeaux and Duraisingh, 2007; Issar et al., 2009; Trelle et al., 2009; Crowley et al., 2011; Kaur et al., 2016; Saraf et al., 2016; Coetzee et al., 2017; Gomez-Diaz et al., 2017; Gupta and Bozdech, 2017; Gupta et al., 2017; Ngwa et al., 2017; Herrera-Solorio et al., 2019; Witmer et al., 2020; Connacher et al., 2021; Kumar et al., 2021; Ngwa et al., 2021). Here, we review the current knowledge regarding epigenetic regulation in *P. falciparum* parasites with a specific focus on the recent developments in our understanding of how histone PTM landscapes orchestrate life cycle progression.

## *P. Falciparum* Parasite Life Cycle

**FIGURE 1:**
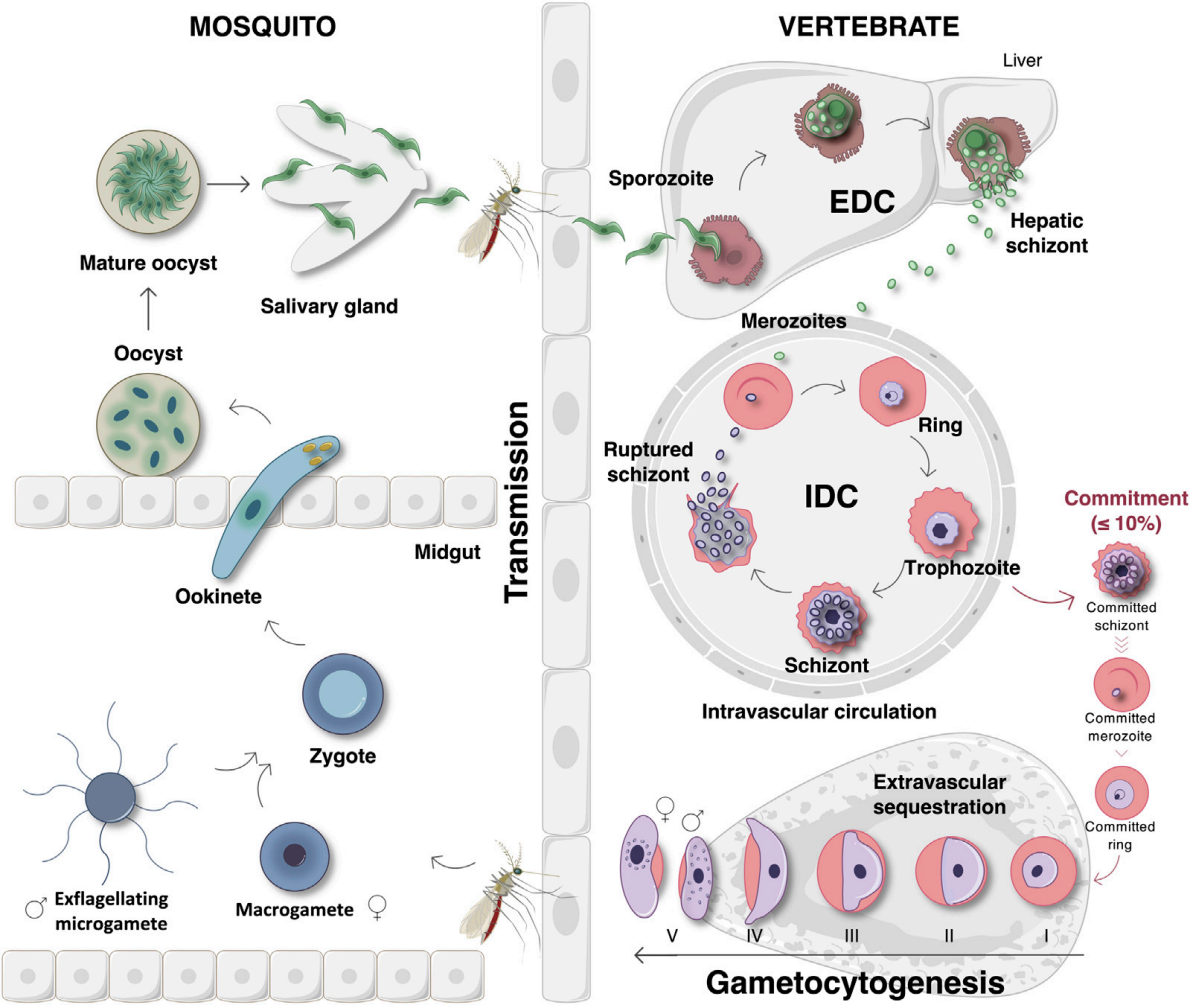
The life cycle of *P. falciparum* parasites. The life cycle begins when an *Anopheles* mosquito vector injects sporozoites into the circulatory system of a human host. The sporozoites then travel to and invade liver cells to initiate the exoerythrocytic developmental cycle (EDC) that results in the formation hepatic schizonts. Once mature, these hepatic schizonts release merozoites into the peripheral circulation. Merozoites invade erythrocytes and initiate the 48-hour intraerythrocytic developmental cycle (IDC) that involves asexual proliferation through ring, trophozoite, and schizont stages. Schizonts then rupture and release new daughter merozoites that repeat this cycle once again. Within each IDC, a small proportion (≤10%) of the parasites will deviate from this fate and instead commit to gametocytogenesis. Sexually committed merozoites invade erythrocytes, forming stage I gametocytes that sequester in the bone marrow where subsequent maturation into gametocyte stages II–IV occurs. Gametocytogenesis yields mature stage V gametocytes that re-enter into the host’s circulatory system where they are ideally situated for transmission to the mosquito during feeding. Once taken up during a blood meal, gametogenesis ensues in the mosquito midgut in which female macrogametes are formed and male gametocytes undergo exflagellation to form microgametes. Thereafter, the microgametes fertilize macrogametes, forming diploid zygotes. Zygote development involves maturation into an ookinete and then an oocyst that contains new, maturing sporozoites. Finally, the mature oocysts rupture, releasing sporozoites that travel to the mosquito’s salivary glands where they will be transmitted to a new host during feeding. Image was created with BioRender.com.

Malaria infections are initiated after sporozoite infection of liver cells. After exoerythrocytic development (EDC), merozoites are released into the peripheral circulation to invade erythrocytes, initiating a new asexual intraerythrocytic developmental cycle (IDC) (Figure 1) (Kappe et al., 2004). Here, ring, trophozoite and schizont development leads to population expansion and the release of new daughter merozoites to repeat the cycle (Dvorak et al., 1975). Parasite survival and transmission are ensured by the divergence of a small proportion (≤10%) of the parasites that commit to sexual differentiation. In *P. falciparum*, this process is uniquely associated with stage differentiation with stage I gametocytes sequestering in the bone marrow to maturate into stages II-IV (Figure 1) (Sinden et al., 1978; Carter and Graves, 1988; Aguilar et al., 2014; Joice et al., 2014). This process of gametocytogenesis yields mature stage V male and female gametocytes that re-enter the host’s circulatory system for transmission to female *Anopheles* mosquitoes during feeding (Smalley et al., 1981; Aguilar et al., 2014). Since stage V gametocytes are the only transmissible forms of the malaria parasite, they have become the focus of the discovery and development of transmission-blocking compounds to aid in malaria elimination strategies (Delves, 2012; Birkholtz et al., 2016). Following gametogenesis of micro- (male) and macro- (female) gametocytes in the mosquito midgut, haploid gametes are fertilized to produce a diploid zygote that transforms into a tetraploid motile ookinete (Figure 1). The maturation of ookinetes into oocysts results in the formation of sporozoites, which can be transmitted to a new host during feeding (Sinden et al., 1978; Billker et al., 1998).

## Nucleosome and Chromatin Structure in *P. Falciparum*

**FIGURE 2:**
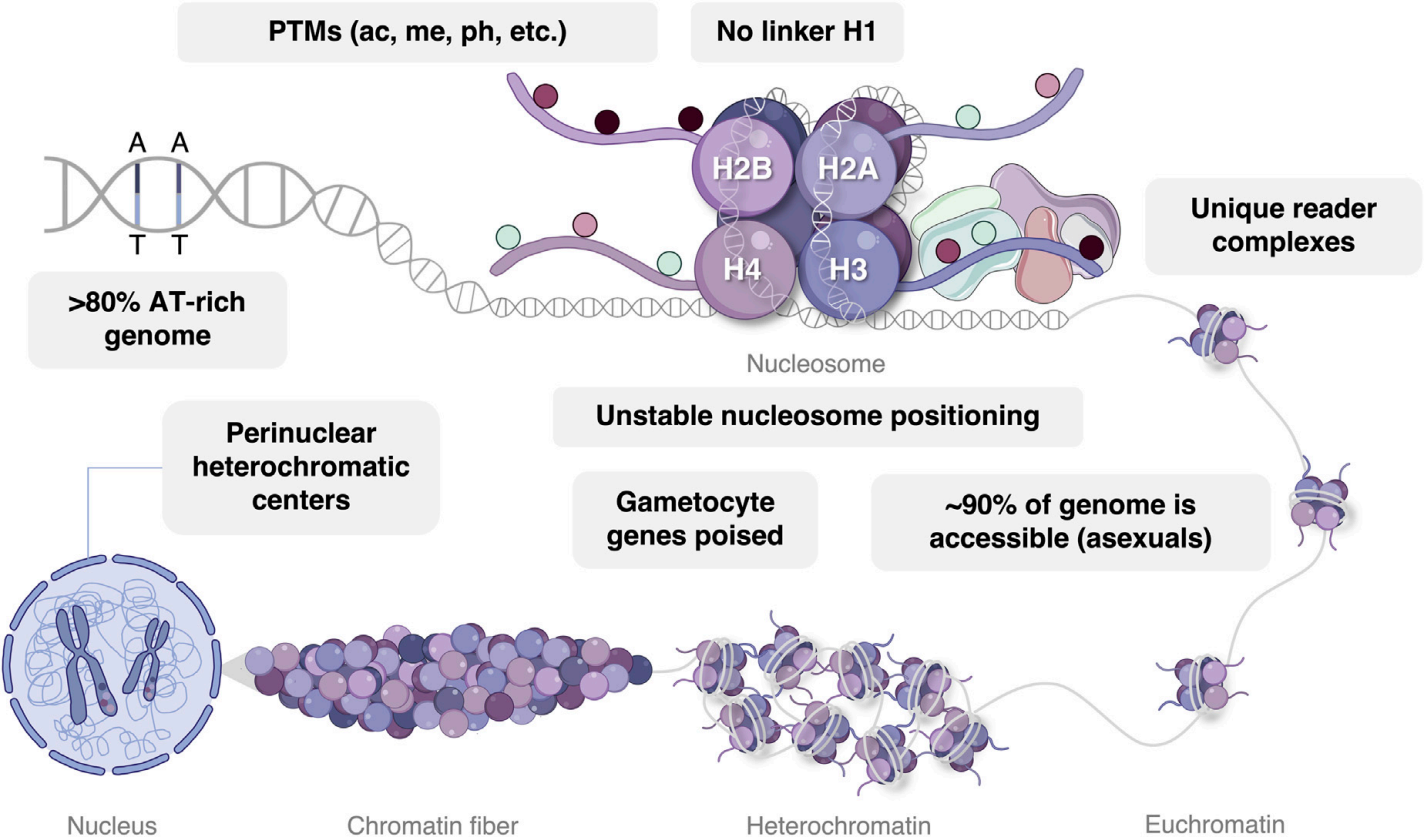
Epigenetic gene regulation in *P. falciparum* parasites. DNA with an AT bias is wrapped around a nucleosome with conventional eukaryotic histone proteins, including H2A, H2B, H3, and H4. The majority of histone post-translational modifications (PTMs) are read by unique epigenetic reader complexes consisting of somewhat divergent writer and eraser enzymes. In asexual parasites, the majority of chromatin exists in an euchromatic conformational state, while gametocyte chromatin exhibits an expansion of H3K9me3-mediated heterochromatin and is stage-specifically poised for transcriptional activation. Perinuclear heterochromatic centers reside in the nucleus associated with *var* gene regulation. Image was created with BioRender.com.

The *P. falciparum* nucleosome consists of 147 bp of DNA associated with an octamer of four histone proteins, typically the H2A, H2B, H3, and H4 core histones, each of which display particular functional and stage-specific characteristics (Figure 2) (Miao et al., 2006; Trelle et al., 2009). Within certain nucleosomes, these core histones may be exchanged for their variants, namely, H2A.Z, H2B.Z, H3.3, and H3Cen, which may also be modified by an array of chemical groups. These variant histones are typically incorporated to demarcate or influence the properties of specific chromatin domains. For example, distinct intergenic regions of the *P. falciparum* genome are demarcated by double-variant nucleosomes containing H2A.Z and H2B.Z while H3.3 is incorporated to index euchromatic coding regions and subtelomeric repeat regions (Bartfai et al., 2010; Hoeijmakers et al., 2013; Petter et al., 2013; Fraschka et al., 2016). Despite sequence divergence, *P. falciparum* histones have not adapted increased binding affinity for the extremely A + T-rich (∼80%) genome (Gardner et al., 2002; Silberhorn et al., 2016). Combined with the apicomplexan-specific absence of the H1 linker histone (Gardner et al., 2002; Sullivan et al., 2006) and short nucleosome repeat length (155 bp) (Silberhorn et al., 2016), this evolution results in a general lack of stable nucleosome patterns in asexual *P. falciparum* parasites (Hoeijmakers et al., 2012). Except for a few internal gene clusters that are brought into proximity with subtelomeric heterochromatin *via* chromatin looping, typical eukaryotic topologically associating domains are therefore largely absent in *P. falciparum* parasites (Lemieux et al., 2013; Bunnik et al., 2014; Bunnik et al., 2018)*.*

Three-dimensional chromatin structure influences the accessibility of DNA to chromatin-binding proteins and transcription factors, thereby enhancing or repressing gene expression (Zheng and Xie, 2019). As such, the rearrangement of genome spatial organization occurs throughout the parasite’s life cycle and corresponds with changes in transcriptional activity (Ay et al., 2014; Bunnik et al., 2014). A relatively simple and dichotomous nuclear organization characterizes the asexual parasite with the majority of the genome (∼90%) in a euchromatic state and with the remainder of the DNA present in perinuclear heterochromatic centers (Hoeijmakers et al., 2012; Brancucci et al., 2014; Coleman et al., 2014). Trophozoites exhibit the most euchromatic and accessible genome organization, in line with the increased level of transcriptional activity required in preparation of schizogony (Ay et al., 2014). Contrasting with this restricted distribution of heterochromatin in the asexual parasite stages, repressive factors become more prolific in the gametocyte stages (Brancucci et al., 2014; Coetzee et al., 2017; Bunnik et al., 2018; Fraschka et al., 2018), reflecting the specific transcriptional environment that underlies sexual differentiation and development (Poran et al., 2017; Walzer et al., 2018; van Biljon et al., 2019).

Chromatin status is largely influenced by post-translational modification of the N-terminal tails of histone proteins that make up the core of the nucleosome (Goll and Bestor, 2002). Quantitative mass spectrometry and antibody-based techniques such as Western blotting has been used to identify histone PTMs in *P. falciparum* parasites with acetylated, methylated, phosphorylated, ubiquitinated, and SUMOylated histones quantitatively detected throughout the life cycle (Miao et al., 2006; Salcedo-Amaya et al., 2009; Trelle et al., 2009; Treeck et al., 2011; Dastidar et al., 2013; Gupta et al., 2013; Ukaegbu et al., 2014; Cobbold et al., 2016; Kaur et al., 2016; Saraf et al., 2016; Coetzee et al., 2017; Gupta et al., 2017; Bui et al., 2019; Green et al., 2020). Acetylation and methylation marks indeed form the major component of histone PTMs in *P. falciparum* and have been accurately detected and quantified over multiple life cycle stages. This provides confidence as to their biological relevance, and indeed, a large number of these marks have been extensively functionally validated. The same is not true for the less prevalent marks, particularly for crotonylation and formylation. Quantitative detection of less abundant (or perhaps time-point specific) histone PTMs, including crotonylation, require antibody-based enrichment of histone PTMs prior to mass spectrometric analysis where, for example, H2AK3crK5cr, H2BK18cr, and H4K78cr was identified in *P. falciparum* asexual parasites (Wang and Zhang, 2020). However, besides detection, no information on the importance of crotonylation to parasite development is currently available and it remains to be seen if this histone PTM is functionally distinct from acetylation in the parasite. Remarkably, lysine crotonylation catalyzed by p300 increased transcriptional activation more potently than lysine acetylation in a cell-free system (Sabari et al., 2015) but such functional validation of a biological role for this histone PTM in *Plasmodium* is lacking.

**FIGURE 3:**
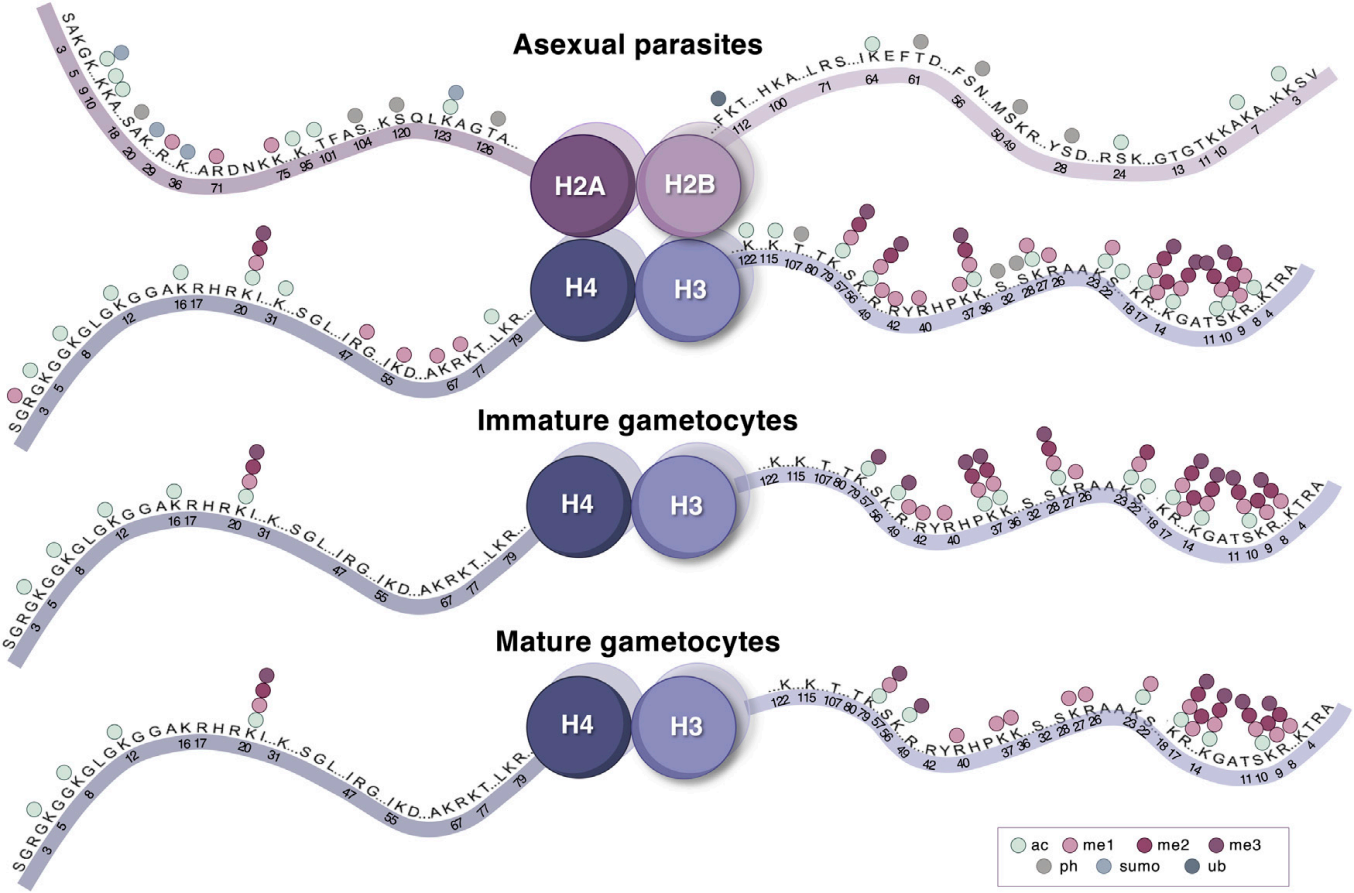
The histone PTM landscape of *P. falciparum* parasites. Histone PTMs (acetylation, different levels of methylation, phosphorylation, SUMOylation, and ubiquitination) are indicated on all positions for the four histones where quantitative detection has been described. This is presented across three life cycle stages—asexual parasites, immature and mature gametocytes. Image was created with BioRender.com.

In addition to individual PTMs, histones of mammalian cells for example are also readily modified with distinct patterns of co-existing PTMs at multiple sites, e.g., methylation of lysine 4 with lysine 9 acetylation of histone H3 (Muller and Muir, 2015). While the functional relevance of a relatively small number of single histone PTMs is well documented, evidence indicating that histone PTMs act in concert with one another to regulate transcriptional programs suggests that combinatorial histone PTMs landscapes contribute an additional layer of regulation in eukaryotes (Berger, 2002; Zhao and Garcia, 2015). The compendium of co-existing histone PTMs on specifically histone H3 and H3.3 in *P. falciparum* parasites was recently updated and revealed that PTM combinations are prevalent in asexual parasites and gametocytes and are considerably diverse and stage-stratified, as determined by quantitative mass spectrometric analysis of whole histone N-terminal tails (von Grüning et al., 2022). Histone PTM crosstalk influences the addition, removal, and binding of effector proteins between histone PTMs in combination (Hunter, 2007). While the enzymes responsible for depositing and removing these individual and combinatorial PTMs are somewhat divergent from other organisms based on homology (Miao et al., 2006; Cui et al., 2008a; Miao et al., 2010a), *P. falciparum* parasites employ a typical eukaryotic histone PTMs repertoire (Miao et al., 2006; Chung et al., 2009; Saraf et al., 2016; Coetzee et al., 2017), including functional acetylation, methylation and phosphorylation (Figure 3) to expand the regulatory capacity of nucleosomes using a limited set of effector proteins (summarized in Table 1).

**TABLE 1:** Catalogue of certain classes of chromatin-associated proteins in *P. falciparum*.

Protein	Gene ID	Essentiality^a^	PTM	Note^b^	Ref
Writers					
Histone lysine methyltransferases (HKMTs)					
PfSET1	PF3D7_0629700	Dispensable (Pf)	H3K4	PbSET1 methylate H4R3, H3K4me3 enriched at intergenic regions	Cui et al. (2008a)
PfSET2 (SETvs)	PF3D7_1322100	Dispensable (Pf)	H3K36	*var* gene silencing	Jiang et al. (2013); Ukaegbu et al. (2014)
PfSET3 (G9a)	PF3D7_0827800	Essential (Pb, Pf)	H3K9		Lopez-Rubio et al. (2009); Volz et al. (2010)
PfSET4	PF3D7_0910000	Dispensable (Pb, Pf)	H3K4	H3K4me3 enriched at intergenic regions	Cui et al. (2008a); Volz et al. (2010)
PfSET5	PF3D7_1214200	Dispensable (Pf)	H3K5/K8/K12		Cui et al. (2008a); Volz et al. (2010)
PfSET6	PF3D7_1355300	Essential (Pf)	H3K4		Cui et al. (2008a); Volz et al. (2010)
PfSET7	PF3D7_1115200	Dispensable (Pb, Pf)	H3K4, H3K9, H3K27		Chen et al. (2016)
PfSET8	PF3D7_0403900	Dispensable (Pb, Pf)	H4K20	H4K20me1/me2/me3; PbSET8 methylate H4R23, H3K59	Cui et al. (2008a); Volz et al. (2010)
PfSET9	PF3D7_0508100	Essential (Pb, Pf)	H4K20		Cui et al. (2008a)
PfSET10	PF3D7_1221000	Dispensable (Pf)	H3K4	H3K4me1, H3K4me2; H3K4me3 enriched at intergenic regions	Volz et al. (2012)
					Ngwa et al. (2021)
** Histone acetyltransferases (HATs)**					
PfGCN5	PF3D7_0823300	Defective (Pf)	H3K9, H3K14	Also have a bromodomain, active *var* gene	Fan et al. (2004b)
PfHAT1	PF3D7_0416400				Cui et al. (2008a)
PfMYST	PF3D7_1118600	Essential (Pb, Pf)	H4K5/K8/K12/K16		Miao et al. (2010a)
** Protein arginine methyltransferase (PRMT)**					
PfPRMT1	PF3D7_1426200	Dispensable (Pb, Pf)	H3R3	Putative class I, H4R3me	Fan et al. (2009)
PfPRMT4/CARM1	PF3D7_0811500	Essential (Pb, Pf)		Putative class I	
PfPRMT5	PF3D7_1361000	Dispensable (Pf)		Putative class II	
**Erasers**					
** Histone demethylases (HDMs)**					
PfJmjC1	PF3D7_0809900	Dispensable (Pf)	H3K9, H3K36		Cui et al. (2008b); Volz et al. (2010)
PfJmjC2	PF3D7_0602800	Dispensable (Pb, Pf)			Cui et al. (2008b); Volz et al. (2010)
PfJmj3	PF3D7_1122200	Dispensable (Pf)	H3K36		Cui et al. (2008b); Matthews et al. (2020)
PfLSD1	PF3D7_1211600	Dispensable (Pb, Pf)			Cui et al. (2008b); Volz et al. (2010)
**Histone deacetylases (HDACs)**					
PfSIR2A	PF3D7_1328800	Dispensable (Pb, Pf)		Transcriptional regulatory protein involved in telomere maintenance and regulation of *var* gene expression	Merrick and Duraisingh. (2010); Mancio-Silva et al. (2013)
PfSIR2B	PF3D7_1451400	Dispensable (Pb, Pf)		Transcriptional regulatory protein involved in telomere maintenance and regulation of *var* gene expression	Petter et al. (2011)
PfHDAC1	PF3D7_0925700	Essential (Pb, Pf)		Class I histone deacetylase 1	Chaal et al. (2010)
PfHDAC2	PF3D7_1008000	Essential (Pb, Pf)		Class II histone deacetylase 2	Coleman et al. (2014)
PfHDAC3/PfHDA2	PF3D7_1472200	Essential (Pf)		Class II histone deacetylase, putative, *var* gene silencing and sexual differentiation	Chaal et al. (2010)
**Readers**					
** Bromodomains: Acetylated histone binding**					
PfSET1	PF3D7_0629700	Dispensable (Pf)	H3K4	Also has PHD-finger domain	Cui et al. (2008b)
PfGCN5	PF3D7_0823300	Defective (Pf) Dispensable (Pb)	H3K9/K14ac	Also has HAT domain, forms part of SAGA-like complex that can bind H3K4me3 *via* a PHD-domain	Hoeijmakers et al. (2019); Miao et al. (2021)
PfBDP1	PF3D7_1033700	Essential (Pb, Pf)	H2B.ZK3/K8/K13/K14/K18ac H3K18/K27ac, H4K5/K8/K12ac	Erythrocyte invasion	Josling and Llinas. (2015); Hoeijmakers et al. (2019)
PfBDP2	PF3D7_1212900	Essential (Pb, Pf)	H2B.ZK3/K8/K13/K14/K18ac H4K5/K8/K12ac		Josling and Llinas. (2015); Hoeijmakers et al. (2019)
PfBDP3	PF3D7_0110500	Dispensable (Pf)			Fleck et al. (2021)
PfBDP4	PF3D7_1475600	Defective (Pf)			Fleck et al. (2021)
PfBDP5/TAF1	PF3D7_1234100	Essential (Pf)			Fleck et al. (2021)
PfBDP6/TAF2	PF3D7_0724700	Essential (Pf)			Fleck et al. (2021)
** PHD-finger/ZF-CW domain: Methylated and acetylated histone binding**					
PfSET1	PF3D7_0629700	Dispensable (Pf)	H3K4		Cui et al. (2008a)
PfSET2	PF3D7_1322100	Dispensable (Pf)			Jiang et al. (2013); Ukaegbu et al. (2014)
PfSET10	PF3D7_1221000	Dispensable (Pf)			Ngwa et al. (2021)
PfSUMO ligase	PF3D7_1360700	Essential (Pb, Pf)			Reiter et al. (2016)
PfLSD1	PF3D7_1211600	Dispensable (Pb, Pf)			Kaur et al. (2016)
PHD finger containing protein	PF3D7_0310200	Dispensable (Pf)			Cowell et al. (2018)
PfPHD1	PF3D7_1008100	Defective (Pf) Dispensable (Pf)	H3K9ac, H3K4me2/me3	Forms part of SAGA-like complex	Miao et al. (2010a); Hoeijmakers et al. (2019)
EELM2 domain containing	PF3D7_1141800	Dispensable (Pf)	H2B.ZK13/K14/K18ac		Hoeijmakers et al. (2019)
PfPHD2	PF3D7_1433400	Essential (Pb, Pf)			Miao et al. (2010a); Hoeijmakers et al. (2019)
PfZFP	PF3D7_0420000	Dispensable (Pf)			Ngwa et al. (2021)
** Chromodomain-like: Methylated histone binding**					
PfHP1	PF3D7_1220900	Essential (Pb, Pf)	H3K9me3	Sexual differentiation, regulation of *var* family	Flueck et al. (2009); Perez-Toledo et al. (2009)
PfMYST	PF3D7_1118600	Essential (Pb, Pf)		Also has HAT and ZnF_C2H2 domains, occupy *var* gene promoter, intergenic regions	Miao et al. (2010b)
Chromo-domain protein	PF3D7_1140700	Dispensable (Pf)			Hoeijmakers et al. (2019)
PfCHD1	PF3D7_1023900	Dispensable (Pf)	H3K9me3	chromodomain-helicase-DNA-binding protein 1 homolog, putative	Watzlowik et al. (2021)

a Essentiality/dispensable nature as indicated by Phenoplasm: http://phenoplasm.org/(Sanderson and Rayner, 2017) or https://www.sanger.ac.uk/group/plasmogem/(Gomes et al., 2015), with a focus on *P. falciparum* data.

Pf = *P. falciparum;* Pb *=* the rodent malaria parasite *P. berghei*.

## Histone Modifying Enzymes in *P. Falciparum*

The methylation of histones in *P. falciparum* is mediated by ten histone methyltransferases (HMTs), all of which belong to the evolutionarily conserved SET [Su (var)3-9, Enhancer of Zeste and Trithorax] domain-containing protein family (Cui et al., 2008a). The site specificities of all, except two (SET5 and SET9) of these HMTs have been determined through recombinant protein expression, pull-down proteomics, mutagenesis, and histone lysine methyltransferase (Cui et al., 2008a; Volz et al., 2012; Jiang et al., 2013; Chen et al., 2016; Ngwa et al., 2021) (Table 1). Less is known regarding the three Jumonji-C (JmjC) domain-containing histone demethylases (HDMs) and the one lysine-specific demethylase 1 (LSD1) that demethylate histones in *P. falciparum,* however, JMJC1 and JMJ3 have predicted and known H3K36-specificity (Cui et al., 2008a; Matthews et al., 2020). Although the precise mechanisms by which histone methyltransferases and demethylases modify histones remain relatively unclear, several studies have demonstrated their importance for asexual and sexual stage development through genetic disruption and chemical interrogation (Jiang et al., 2013; Ukaegbu et al., 2014; Zhang et al., 2018; Coetzee et al., 2020; Matthews et al., 2020; Connacher et al., 2021; Reader et al., 2021) (Table 1).

Histone acetylation is regulated by four histone acetyltransferases (HATs) including proteins from the MYST and GNAT families (Cui et al., 2008a). The acetyltransferase activities of GCN5 and MYST are specific to the lysine residues of H3 and H4, respectively (Cui et al., 2007b; Miao et al., 2010a), with the functional importance of these enzymes evident from the transcriptional deregulation arising from their genetic and chemical disruption (Cui et al., 2007b; Cui et al., 2008b; Chaal et al., 2010; Bhowmick et al., 2020; Miao et al., 2021) (Table 1). Acetyltransferase activities are antagonized by a repertoire of three histone deacetylases (HDACs) and two sirtuin histone deacetylase proteins in *P. falciparum* (Kanyal et al., 2018). Interestingly, the *P. falciparum* genome encodes for an essential YEATS-domain protein (PF3D7_0807000). Its orthologues in mammalians and yeast are readers of crotonylation. It would therefore be interesting to see if similar activity is present in Plasmodia and if so, crotonylation could be important to biological processes in the parasite despite it being a rarely identified PTM (Table 1).

The chromatin reorganization induced by these enzymes results either from a direct physical change in nucleosome structure or the recruitment of epigenetic “reader complexes” that mediate subsequent chromatin remodeling (Latchman, 2010; Abel and Le Roch, 2019) (Table 1). To date, five putative reader complexes have been identified in *P. falciparum* parasites, however, the vast majority remain to be functionally validated (Hoeijmakers et al., 2019). These *P. falciparum* readers largely exist in complexes with other reader proteins and/or transcription factors that would presumably require sequential binding occurrences for recruitment (Hoeijmakers et al., 2019). Such cooperation between epigenetic regulatory proteins is exemplified in the recruitment of the GCN5/ADA2 reader complex by H3K4me3 *via* interaction with PHD1 (Hoeijmakers et al., 2019). Additionally, evidence suggests that as in other organisms, epigenetic complexes in *P falciparum* may be recruited to or “flavored” to specific histone PTMs or combinations of PTMs, as determined by peptide pull-down coupled with mass spectrometry. For example, GCN5/ADA2 core reader complex has at least two distinct “flavors” with a unique composition of reader proteins, such as the presence or absence of PHD1 or PHD2 (Hoeijmakers et al., 2019; Miao et al., 2021). Additionally, the intricate nature of multiprotein reader complexes supports the existence of unique sets of functional histone PTMs combinations in *P. falciparum* parasites whereby different reader proteins bind to PTMs using reader domains. Whether these modifications directly influence chromatin structure via the recruitment of such readers or by virtue of their biochemical properties, HMs are undoubtedly at the core of epigenetic regulation.

## Histone PTM Landscapes in *P. Falciparum*

Although up to 230 histone PTMs have been identified in various qualitative and quantitative proteomic based approaches *P. falciparum* parasites (Salcedo-Amaya et al., 2009; Trelle et al., 2009; Saraf et al., 2016), only 46 of these have been quantitatively validated (Coetzee et al., 2017) (Figure 3). The *P. falciparum* histone PTM repertoire expands beyond the typical eukaryotic histone modifications (e.g., H3K4me3, H3K9me3, and H3K36me3) and contains several less common modifications, including H4K20ac, H3.3K79me3, and H3K23me1 (Coetzee et al., 2017). Similar to the hierarchical cascades of gene expression that are associated with each asexual and sexual stage (Bozdech et al., 2003; van Biljon et al., 2019), strikingly unique and stage-specific histone PTM landscapes characterize each stage of the *P. falciparum* parasite life cycle (Figure 3) (Coetzee et al., 2017).

### Asexual Blood Stage PTM Landscape

The greatest proportion of histone PTMs in the asexual parasite stages consists of acetylation and mono-methylation, both of which are associated with transcriptionally permissive chromatin (Saraf et al., 2016; Coetzee et al., 2017). Throughout the IDC, the mostly euchromatic nature of the genome is marked by nucleosomes containing acetylated histones H3 (K9, K14, and K56) and H4 (K5, K8, K12, and K16) in addition to H3K4me3 and H4K20me1, most of which are positively correlated with gene expression in *P. falciparum* (Gupta et al., 2013). The large transcriptional disruption (∼60% of the genome) and effects on growth that arise from inhibiting histone acetylation and methylation in *P. falciparum* parasites, highlight the crucial nature of these histone PTMs for parasite regulation (Cui et al., 2007a; Cui et al., 2008b; Matthews et al., 2020; Connacher et al., 2021). Regarding combinatorial histone PTMs in the asexual parasite stages, co-existing H3K4me3, H3K9ac, and H3K14ac on histone H3 are achieved through the individual actions of PfGCN5 and PfSET7 (Fan et al., 2004a; Fan et al., 2004b; Salcedo-Amaya et al., 2009; Cui and Miao, 2010; Chen et al., 2016).

Throughout the asexual life cycle, dynamic patterns of H3K18ac and H3K27ac mark the transcriptional start sites of active genes with the AP2-I transcription factor and bromodomain protein 1 (BDP1) reader protein exhibiting similar temporal enrichment at these sites as determined by ChIP-seq and RNA-seq (Tang et al., 2020). Ring and schizonts stages have increased abundance of H4K20me1 and H4K8ac, respectively while acetylation of H3K9 and H3K14 become highly abundant in trophozoites (Gupta et al., 2013; Coetzee et al., 2017). H3K37me1, a novel histone PTMs in *P. falciparum* which has been associated with licensing of replication origins in yeast (Santos-Rosa et al., 2021; von Grüning et al., 2022), is prominent in trophozoite stages. Similarly, on histone H3.3, K37me3 steadily increases in abundance from asexual parasites and immature gametocytes to mature gametocytes (von Grüning et al., 2022). Interestingly, the methylation of H3R17 and H3R40 is more prevalent compared to H3K4me3, suggesting that these histone PTMs may be additional to the canonical acetylation modifications (e.g., H3K9a and H3K14ac) that are involved in transcriptional activation in trophozoites (von Grüning et al., 2022). In addition to this, the connectivity between histone PTMs is exemplified by H3K9acK14ac that is present, and together with the binding of effector proteins, likely contributes to the euchromatic nature of the trophozoite genome (von Grüning et al., 2022). H3K9ac also co-exists with H3K36me3 and exhibits the most notable crosstalk in the trophozoite stages, highlighting the exclusive nature of these particular combinations for asexual parasites, evidenced by quantitative middle-down mass spectrometry (von Grüning et al., 2022). Overall, PTMs on variant histones have also been identified, yet not reproducibly quantified (Saraf et al., 2016; Coetzee et al., 2017). Nonetheless, the C-terminal SQ motif of histone H2A has been shown to be phosphorylated in response to DNA damage (Goyal et al., 2021) in addition to the qualitative detection of serine phosphorylation and lysine acetylation (Coetzee et al., 2017). Similarly, lysine acetylation and methylation, and serine phosphorylation, have also been detected on H3.3, and variant histones H2A.Z, H2B.Z, and centromeric H3 (Saraf et al., 2016; Coetzee et al., 2017; von Grüning et al., 2022). SUMOylation, a PTM that is linked to antagonizing transcription and chromatin remodeling, has been predicted on a combined ten lysine sites on the N-termini of histone H2A and H2A.Z, with histone H4 being a putative target for SUMOylation as well (Issar et al., 2008; Ryu and Hochstrasser, 2021). This suggests that SUMOylation might well be an integral part of the histone PTM language of the parasite. Together with the abundance of individual activating modifications, these combinations provide a critical roadmap that underpins the “just-in-time” transcription of the asexual stages. Interestingly, another ApiAP2 transcription factor family member that is thought to act downstream of AP2-G, AP2-G2, occupancy is highly correlated with repressive H3K36me3, H4K20me3 and H3K27me3 in asexual parasites as determined by pairwise Pearson correlation comparing read counts (Singh et al., 2020). Although AP2-G2 is not essential for asexual proliferation, it is, however, essential for gametocyte maturation where AP2-G2 functions by repressing genes necessary for asexual replication (Singh et al., 2020). Together, this indicates that although present at lower abundance, repressive PTMs are an equally important facet of transcriptional regulation during asexual proliferation. Interestingly, negative crosstalk has been observed between the novel arginine PTMs, H3R40me1, and H3R17me1 in the trophozoite stages (von Grüning et al., 2022). It remains to be seen if H3R40me1 is important to microgametogenesis, similar to its involvement in sporulation in yeast, a process similar to male gametogenesis (Govin et al., 2010) and if H3R17me1 serves independently from canonical euchromatic PTMs in trophozoites to de-represses silenced chromatin as in mammalian cells (Miller and Grant, 2013). In model organisms, H3K56me1&2 have been implicated as docking sites for DNA replication and repair enzymes (Lee et al., 2012; Yu et al., 2012; Janssen et al., 2019), inviting the question as to whether the peak abundance of these histone PTMs in the ring-stage parasites may be indicative of the parasite’s preparation for genome replication in the proceeding stages.

The most notable increase in stage-specific histone PTM abundance is present during the schizont stages (Coetzee et al., 2017), specifically for H3K4ac and H4K16ac, both of which have roles in transcriptional activation and is therefore unsurprising given the increased metabolic and transcriptional activity in schizonts (Bozdech et al., 2003; van Biljon et al., 2019). H3K4ac is abundant in active promoter regions with the levels of this PTM regulated by H3K4 methylation in higher eukaryotes (Guillemette et al., 2011). In line with this, H3K4me2&3 are also present in high abundance in schizonts (Coetzee et al., 2017), with functional data indicating H3K4 methylation also positively influences transcription via the recruitment of the GCN5/ADA2 and the associated BDP1/2 reader complexes (Fan et al., 2004a; Hoeijmakers et al., 2019). Although the roles of H4K16ac are less clear, studies in other organisms have documented this PTM as a high-affinity docking site for chromatin-remodeling complexes that promote transcription (Ruthenburg et al., 2011), with the abundance of H4K16ac in asexual parasites, therefore, presenting a possible core mechanism that promotes euchromatin formation. H4K20me3 also exhibits a schizont stage-specific peak in abundance (Coetzee et al., 2017) and was recently identified by machine learning algorithms to be highly indicative of active gene expression in *P. falciparum* parasites (Read et al., 2019).

### Histone PTMs That Regulate Clonally Variant Gene Expression and Phenotypic Plasticity

The involvement of epigenetic regulatory mechanisms in variant expression of several gene families have been extensively studied in *P. falciparum*. Epigenetic regulation of processes such as immune evasion (e.g., *var*, *rifin*, and *stevor*), erythrocyte remodeling (e.g., *PfMC-2TM*) and lipid metabolism (e.g., acyl-CoA binding proteins) (Rovira-Graells et al., 2012; Gomez-Diaz et al., 2017) ensures heritability and rapid adaptation in response to environmental changes. For example, single *var* gene expression is ensured by H2A.Z/H2B.Z double-variant histones, H3.3 and H3K9ac occupancy (Petter et al., 2013), and heritable transmission is ensured by a poised state associated with H3K4me2 (Volz et al., 2012). Additionally, H3.3 is also present in the promoter region and has thus been proposed to be involved in regulating the single active or poised *var* gene (Fraschka et al., 2016). The remainder of the *var* gene family in perinuclear repressive centers is actively silenced with H3K9me3/HP1 and H3K36me3 (Chookajorn et al., 2007; Flueck et al., 2009; Lopez-Rubio et al., 2009; Salcedo-Amaya et al., 2009; Trelle et al., 2009; Jiang et al., 2013; Ay et al., 2014; Ukaegbu et al., 2014; Bunnik et al., 2019). H3K9me3/HP1 have similar strong mechanistic links with the regulation of the *rif*, *stevor*, *pfmc-2tm*, and lysophospholipase families as well as genes that direct the transition from asexual proliferation to sexual differentiation in *P. falciparum* parasites (Rovira-Graells et al., 2012; Brancucci et al., 2014; Gomez-Diaz et al., 2017).

### Histone PTMs That Orchestrate the Asexual to Sexual Stage Transition

Several extrinsic drivers of the switch from proliferation to sexual differentiation in *P. falciparum* have been investigated including increased parasitaemia, antimalarial drugs, and lysophosphatidylcholine (lysoPC) depletion (Trager and Gill, 1992; Buckling et al., 1999; Williams, 1999; Dyer and Day, 2000; Brancucci et al., 2017). Currently, the specific mechanism linking these factors with the switch to sexual differentiation is unclear. Nevertheless, several essential genetic factors have reproducibly been shown to be upregulated upon this cue and drive sexual differentiation in *Plasmodium* parasites (Kafsack et al., 2014; Sinha et al., 2014). The commitment process is largely driven by AP2-G, a member of the apicomplexan-specific Apetala2 (ApiAP2) DNA binding domain-containing protein family (Balaji et al., 2005; Kafsack et al., 2014). During the IDC, the *ap2-g* locus remains silenced within H3K9me3/HP1-mediated heterochromatin (Brancucci et al., 2014; Filarsky et al., 2018) and the expression of gametocyte development protein 1 gene (*gdv1*) is inhibited by its own antisense RNA. The gametocyte essential factor, AP2-G5, further prevents sexual commitment by binding *ap2-g* upstream and exonic regions that are responsible for maintaining local heterochromatin which suppresses *ap2-g* gene expression (Shang et al., 2021). Upon the cue for commitment, GDV1 is upregulated and evicts H3K9me3/HP1, resulting in the de-repression of the *ap2-g* locus (Poran et al., 2017; Filarsky et al., 2018). Sequence-specific binding of AP2-G to promoters results the transcriptional activation of hundreds of genes that are predominantly involved in commitment and early-stage gametocyte development, including those encoding invasion proteins that are co-regulated by a second AP2 TF, AP2-I (Kafsack et al., 2014; Poran et al., 2017; Josling et al., 2020). Following sexual commitment, AP2-G5 downregulates AP2-G as well as the early gametocyte genes activated by AP2-G, resulting in a cascade of gene on/off switching that is required for gametocyte maturation (Shang et al., 2021). Aside from the abovementioned H3K9 modifications, the histone PTM landscape dynamics of commitment remain unknown. It is possible that the interaction between H3K9me3 and PfHP1 is influenced by PTMs that co-exist with H3K9me3, most notably, H3S10ph. Although not proven yet, there may be a negative crosstalk relationship between H3K9me3 and H3S10ph in *Plasmodium*, similar to how H3S10ph evicts HP1 from H3K9me3 and prevents the spread of H3K9me2/me3 across the genome in mammalian cells (Fischle et al., 2005; Hirota et al., 2005; Chen et al., 2018). Interestingly, H3.3 carries less PTMs in combination than H3 in trophozoite stages but increase to up to seven PTMs co-existing on the H3.3 histone tail in immature gametocytes and could be linked to the drastic shift in transcriptional program in immature gametocytes (von Grüning et al., 2022).

In model organisms, differentiation consists of multiple stages, each with characteristic epigenome signatures (Bhanu et al., 2016). Elucidating whether the histone PTMs that characterize differentiation initiation in other eukaryotes, such as H3K4 H3K9, H3K20 methylation (Bhanu et al., 2016) and global histone acetylation (Meshorer et al., 2006; Tan et al., 2013), is also associated with sexual commitment in *P. falciparum* remains an interesting question. The impact of the metabolic shift between asexual and gametocytes on the chromatin milieu also warrants clarification, with the indication that loss of function of acetyl-CoA synthetase leads to chromatin hypoacetylation and that the restriction of *S*-adenosyl-L-methionine availability can cause heterochromatin formation (Prata et al., 2021; Summers et al., 2021).

### Histone PTM Landscapes That Direct Gametocytogenesis

The histone PTM landscapes associated with gametocyte development stand in strong contrast with those involved in asexual stage-specific transcriptional regulation (Coetzee et al., 2017) (Figure 3). Gametocytogenesis is characterized by fewer typical euchromatic PTMs and an increased abundance of repressive di- and tri-methylation on histone H3 (Coetzee et al., 2017). The presence of these PTMs is particularly notable in the early (I-III) stages of gametocyte development and include H3K9me3, H3K27me2&3, and H3K36me2 (Coetzee et al., 2017), the latter of which has been linked to global repression in asexual stages (Karmodiya et al., 2015). Other less well-characterized PTMs including H3K37me1, H3R17me1 and H3R17me2 are also prominent in the early-stage gametocytes (von Grüning et al., 2022).

During gametocytogenesis, H3K9me3/HP1 patterns undergo substantial changes, leading to large heterochromatin reorganization events (Bunnik et al., 2018; Fraschka et al., 2018). Together with a subtelomeric stretch of chromosome 10, genes encoding merozoite invasion proteins become strongly associated with the distinct perinuclear H3K9me3/HP1-enriched heterochromatin clusters (Brancucci et al., 2014; Bunnik et al., 2018). Additionally, heterochromatin expansion across the subtelomeric region of chromosome 14 leads to the formation of a topologically associated superdomain (>800 kb) with a strong boundary that is postulated to mediate the expression of cell growth and division inhibitors during gametocyte development (Bunnik et al., 2018), similar to what has been observed in other organisms (Dixon et al., 2012). During gametocytogenesis, certain genomic regions show reduced H3K9me3/HP1 occupancy, including those containing sexual development genes (e.g., *ap2-g*, *pfg14-744*, and *pfg14-748*) (Bunnik et al., 2018; Fraschka et al., 2018).

Transcriptional repression of asexual-stage associated genes that become obsolete in the immature gametocyte stages has been associated with several PTMs including H3K36me2&3 (Connacher et al., 2021). Despite the strong link between H3K36me3 and AP2-G2 occupancy in the asexual stages, fewer H3K36me2&3 enriched genes are associated with AP2-G2 in the early-stage gametocyte, indicating that, similar to the divergent transcriptional programs, regulation by histone PTM landscapes is highly specific to gametocytogenesis. The histone PTM landscape of immature gametocytes is also characterized by an increased abundance of repressive H3K27me2&3, H4K20me3 and H3K27ac, the latter two of which co-occur with AP2-G2 in asexual parasites (Singh et al., 2020). While it is tempting to speculate that these histone PTMs are similarly involved in recruiting transcription factors and chromatin modifying enzymes in *P. falciparum* as in other eukaryotes (Carrozza et al., 2005; Keogh et al., 2005; Maltby et al., 2012; Ren et al., 2021), the functional and mechanistic links between these epigenetic factors remain interesting points for further study.

Arginine methylation combinations may represent a key feature of epigenetic regulation for gametocyte development, exemplified by the exclusive combinations of H3R42me1 with H3K37me1 and H3R40me1 (von Grüning et al., 2022). The abundance of combinatorial H3K14me2 with H3K9me1 or H3K18 methylation offers the first suggestion that co-existing PTMs may also modulate heterochromatin establishment in early-stage gametocytes (von Grüning et al., 2022). PTM crosstalk in the late-stage gametocyte reflects a unique relationship between PTMs compared to other stages of the parasite’s life cycle. Late-stage (stages IV/V) gametocytes are transcriptionally poised for subsequent gamete formation, as reflected by equal distribution of hetero- and euchromatin marks and the use of translational repression mechanisms in female gametocytes (Lasonder et al., 2016; Coetzee et al., 2017). The combination of H3K27me1K36me1 (Coetzee et al., 2017; von Grüning et al., 2022), may further contribute towards generating poised mRNAs, congruent with the dependence of H3K27me1 on H3K36me1 to induce transcription in embryonic stem cells (Jung et al., 2013). Ultimately, the histone PTM landscape of the late-stage gametocytes presents a suitable environment in which certain genes remain transcriptionally poised in preparation for rapid fertilization in the mosquito.

## Conclusion and Future Directions

Given the central nature of histone PTMs for *P. falciparum* reviewed here, further understanding the mechanistic relevance of individual and combinatorial PTMs will help identification of the most suited epigenetic targets for drug-based intervention. Although the stage-specific functional relevance of certain individual histone PTMs has been elucidated, the remaining PTMs and combinations with stage-specific patterns should be investigated. Specifically, the influence of these PTMs on asexual and sexual development and the writers, erasers and readers of the histone PTM landscapes and thus insights into the direct or indirect consequences on transcription. In the future, we foresee the use of the combinatorial histone code as a “barcode” that could define parasite life cycle stages or ongoing biological processes. This barcode is dynamic and associated with remarkable changes to allow shifts in chromatin status in different developmental stages of the parasite. In-depth critical analyses of the available data clearly implicate this changing histone PTM landscape as higher-order mechanisms of regulation of gene expression, providing a clear blueprint associated with required phenotypic changes in the parasite to adapt to host responses (antigenic variation), allow massive population expansion in the host (asexual proliferation) and exploiting host environments to allow differentiation and species continuation (sexual development). However, as histone PTMs are not isolated but co-exist to fine-tune binding of effector proteins, the PTM landscape and combinatorial code may indeed guide a highly specific transcriptional program. Lastly, the cause and/or effect relationship between nutrient metabolism and available substrates to modify the histones requires deconvolution. Taken together, the findings discussed in this necessary update demonstrate that although many factors, including nucleosome positioning, chromatin structure and transcription factors participate in transcriptional regulation in *P. falciparum* parasites, the histone PTM landscapes represent the road maps that are central to the specific biology of each stage. The demonstration of histone PTMs as key determinants of both asexual proliferation and sexual differentiation of *P. falciparum* parasites highlights the individual factors as novel targets for disrupting malaria transmission and underscore the importance of a more thorough understanding of these critical regulators.

## Abbreviations

Ac, acetylation; EDC, exoerythrocytic developmental cycle; gdv1, gametocyte development protein 1; HAT, histone acetyltransferase; HDAC, histone deacetylase; HDM: histone demethylase; histone PTMs, histone post-translational modifications; HMT, histone methyltransferase; HP1, heterochromatin protein 1; IDC, intraerythrocytic developmental cycle; JMJC, jumonji-C containing demethylase; LSD, lysine-specific demethylase; me, methylation.

## References

[B1] Abel S. Le Roch K. G. (2019). The Role of Epigenetics and Chromatin Structure in Transcriptional Regulation in Malaria Parasites. Brief. Funct. Genomics 18, 302–313. 10.1093/bfgp/elz005 31220857 PMC6859822

[B2] Aguilar R. Magallon-Tejada A. Achtman A. H. Moraleda C. Joice R. Cisteró P. (2014). Molecular Evidence for the Localization of *Plasmodium Falciparum* Immature Gametocytes in Bone Marrow. Blood 123, 959–966. 10.1182/blood-2013-08-520767 24335496 PMC4067503

[B3] Ay F. Bunnik E. M. Varoquaux N. Bol S. M. Prudhomme J. Vert J.-P. (2014). Three-dimensional Modeling of the P. Falciparum Genome during the Erythrocytic Cycle Reveals a strong Connection between Genome Architecture and Gene Expression. Genome Res. 24, 974–988. 10.1101/gr.169417.113 24671853 PMC4032861

[B4] Balaji S. Babu M. M. Iyer L. M. Aravind L. (2005). Discovery of the Principal Specific Transcription Factors of Apicomplexa and Their Implication for the Evolution of the AP2-Integrase DNA Binding Domains. Nucleic Acids Res. 33, 3994–4006. 10.1093/nar/gki709 16040597 PMC1178005

[B5] Bártfai R. Hoeijmakers W. A. M. Salcedo-Amaya A. M. Smits A. H. Janssen-Megens E. Kaan A. (2010). H2A.Z Demarcates Intergenic Regions of the *Plasmodium Falciparum* Epigenome that Are Dynamically Marked by H3K9ac and H3K4me3. Plos Pathog. 6, e1001223. 10.1371/journal.ppat.1001223 21187892 PMC3002978

[B6] Berger S. L. (2002). Histone Modifications in Transcriptional Regulation. Curr. Opin. Genet. Dev. 12, 142–148. 10.1016/s0959-437x(02)00279-4 11893486

[B7] Bhanu N. V. Sidoli S. Garcia B. A. (2016). Histone Modification Profiling Reveals Differential Signatures Associated with Human Embryonic Stem Cell Self-Renewal and Differentiation. Proteomics 16, 448–458. 10.1002/pmic.201500231 26631989 PMC4819991

[B8] Bhowmick K. Tehlan A. SunitaSudhakar R. Sudhakar R. Kaur I. Sijwali P. S. (2020). *Plasmodium Falciparum* GCN5 Acetyltransferase Follows a Novel Proteolytic Processing Pathway that Is Essential for its Function. J. Cel Sci 133. 10.1242/jcs.236489 31862795

[B9] Billker O. Lindo V. Panico M. Etienne A. E. Paxton T. Dell A. (1998). Identification of Xanthurenic Acid as the Putative Inducer of Malaria Development in the Mosquito. Nature 392, 289–292. 10.1038/32667 9521324

[B10] Birkholtz L.-M. Coetzer T. L. Mancama D. Leroy D. Alano P. (2016). Discovering New Transmission-Blocking Antimalarial Compounds: Challenges and Opportunities. Trends Parasitol. 32, 669–681. 10.1016/j.pt.2016.04.017 27209388

[B11] Bozdech Z. Llinás M. Pulliam B. L. Wong E. D. Zhu J. Derisi J. L. (2003). The Transcriptome of the Intraerythrocytic Developmental Cycle of *Plasmodium Falciparum* . Plos Biol. 1, E5. 10.1371/journal.pbio.0000005 12929205 PMC176545

[B12] Brancucci N. M. B. Bertschi N. L. Zhu L. Niederwieser I. Chin W. H. Wampfler R. (2014). Heterochromatin Protein 1 Secures Survival and Transmission of Malaria Parasites. Cell Host & Microbe 16, 165–176. 10.1016/j.chom.2014.07.004 25121746

[B13] Brancucci N. M. B. Gerdt J. P. Wang C. De Niz M. Philip N. Adapa S. R. (2017). Lysophosphatidylcholine Regulates Sexual Stage Differentiation in the Human Malaria Parasite *Plasmodium Falciparum* . Cell 171, 1532–1544. 10.1016/j.cell.2017.10.020 29129376 PMC5733390

[B14] Buckling A. Ranford-Cartwright L. C. Miles A. Read A. F. (1999). Chloroquine Increases *Plasmodium Falciparum* Gametocytogenesis *In Vitro* . Parasitology 118 (Pt 4), 339–346. 10.1017/s0031182099003960 10340323

[B15] Bui H. T. N. Niederwieser I. Bird M. J. Dai W. Brancucci N. M. B. Moes S. (2019). Mapping and Functional Analysis of Heterochromatin Protein 1 Phosphorylation in the Malaria Parasite Plasmodium Falciparum. Sci. Rep. 9, 16720. 10.1038/s41598-019-53325-9 31723180 PMC6853920

[B16] Bunnik E. M. Cook K. B. Varoquaux N. Batugedara G. Prudhomme J. Cort A. (2018). Changes in Genome Organization of Parasite-specific Gene Families during the *Plasmodium* Transmission Stages. Nat. Commun. 9, 1910. 10.1038/s41467-018-04295-5 29765020 PMC5954139

[B17] Bunnik E. M. Polishko A. Prudhomme J. Ponts N. Gill S. S. Lonardi S. (2014). DNA-encoded Nucleosome Occupancy Is Associated with Transcription Levels in the Human Malaria Parasite *Plasmodium Falciparum* . BMC Genomics 15, 347. 10.1186/1471-2164-15-347 24885191 PMC4035074

[B18] Bunnik E. M. Venkat A. Shao J. Mcgovern K. E. Batugedara G. Worth D. (2019). Comparative 3D Genome Organization in Apicomplexan Parasites. Proc. Natl. Acad. Sci. USA 116, 3183–3192. 10.1073/pnas.1810815116 30723152 PMC6386730

[B19] Carrozza M. J. Li B. Florens L. Suganuma T. Swanson S. K. Lee K. K. (2005). Histone H3 Methylation by Set2 Directs Deacetylation of Coding Regions by Rpd3S to Suppress Spurious Intragenic Transcription. Cell 123, 581–592. 10.1016/j.cell.2005.10.023 16286007

[B20] Carter R. Graves P. M. (1988). “Gametocytes,” in Malaria: Principles and Practice of Malariology. Editor Mcgregor I. (London: Churchill Livingstone), 253–305.

[B21] Chaal B. K. Gupta A. P. Wastuwidyaningtyas B. D. Luah Y.-H. Bozdech Z. (2010). Histone Deacetylases Play a Major Role in the Transcriptional Regulation of the Plasmodium Falciparum Life Cycle. Plos Pathog. 6, e1000737. 10.1371/journal.ppat.1000737 20107518 PMC2809759

[B22] Chen C. C. L. Goyal P. Karimi M. M. Abildgaard M. H. Kimura H. Lorincz M. C. (2018). H3S10ph Broadly marks Early-Replicating Domains in Interphase ESCs and Shows Reciprocal Antagonism with H3K9me2. Genome Res. 28, 37–51. 10.1101/gr.224717.117 29229671 PMC5749181

[B23] Chen P. B. Ding S. Zanghì G. Soulard V. Dimaggio P. A. Fuchter M. J. (2016). *Plasmodium Falciparum* PfSET7: Enzymatic Characterization and Cellular Localization of a Novel Protein Methyltransferase in Sporozoite, Liver and Erythrocytic Stage Parasites. Sci. Rep. 6, 21802. 10.1038/srep21802 26902486 PMC4763181

[B24] Chookajorn T. Dzikowski R. Frank M. Li F. Jiwani A. Z. Hartl D. L. (2007). Epigenetic Memory at Malaria Virulence Genes. Proc. Natl. Acad. Sci. U.S.A. 104, 899–902. 10.1073/pnas.0609084103 17209011 PMC1764221

[B25] Cobbold S. A. Santos J. M. Ochoa A. Perlman D. H. Llinás M. (2016). Proteome-wide Analysis Reveals Widespread Lysine Acetylation of Major Protein Complexes in the Malaria Parasite. Sci. Rep. 6, 19722. 10.1038/srep19722 26813983 PMC4728587

[B26] Coetzee N. Sidoli S. Van Biljon R. Painter H. Llinás M. Garcia B. A. (2017). Quantitative Chromatin Proteomics Reveals a Dynamic Histone post-translational Modification Landscape that Defines Asexual and Sexual *Plasmodium Falciparum* Parasites. Sci. Rep. 7, 607. 10.1038/s41598-017-00687-7 28377601 PMC5428830

[B27] Coetzee N. von Grüning H. Opperman D. Van Der Watt M. Reader J. Birkholtz L.-M. (2020). Epigenetic Inhibitors Target Multiple Stages of *Plasmodium Falciparum* Parasites. Sci. Rep. 10, 2355. 10.1038/s41598-020-59298-4 32047203 PMC7012883

[B28] Coleman B. I. Skillman K. M. Jiang R. H. Y. Childs L. M. Altenhofen L. M. Ganter M. (2014). A *Plasmodium Falciparum* Histone Deacetylase Regulates Antigenic Variation and Gametocyte Conversion. Cell Host & Microbe 16, 177–186. 10.1016/j.chom.2014.06.014 25121747 PMC4188636

[B29] Comeaux C. A. Duraisingh M. T. (2007). Unravelling a Histone Code for Malaria Virulence. Mol. Microbiol. 66, 1291–1295. 10.1111/j.1365-2958.2007.06038.x 18028316

[B30] Connacher J. Josling G. A. Orchard L. M. Reader J. Llinás M. Birkholtz L.-M. (2021). H3K36 Methylation Reprograms Gene Expression to Drive Early Gametocyte Development in *Plasmodium Falciparum* . Epigenetics & Chromatin 14, 19. 10.1186/s13072-021-00393-9 33794978 PMC8017609

[B31] Cowell A. N. Istvan E. S. Lukens A. K. Gomez-Lorenzo M. G. Vanaerschot M. Sakata-Kato T. (2018). Mapping the Malaria Parasite Druggable Genome by Using *In Vitro* Evolution and Chemogenomics. Science 359, 191–199. 10.1126/science.aan4472 29326268 PMC5925756

[B32] Crowley V. M. Rovira-Graells N. de Pouplana L. R. Cortés A. (2011). Heterochromatin Formation in Bistable Chromatin Domains Controls the Epigenetic Repression of Clonally Variant Plasmodium Falciparum Genes Linked to Erythrocyte Invasion. Mol. Microbiol. 80, 391–406. 10.1111/j.1365-2958.2011.07574.x 21306446

[B33] Cui L. Fan Q. Cui L. Miao J. (2008a). Histone Lysine Methyltransferases and Demethylases in *Plasmodium Falciparum* . Int. J. Parasitol. 38, 1083–1097. 10.1016/j.ijpara.2008.01.002 18299133 PMC4566933

[B34] Cui L. Miao J. (2010). Chromatin-mediated Epigenetic Regulation in the Malaria Parasite *Plasmodium Falciparum* . Eukaryot. Cel 9, 1138–1149. 10.1128/ec.00036-10 PMC2918932 20453074

[B35] Cui L. Miao J. Cui L. (2007a). Cytotoxic Effect of Curcumin on Malaria Parasite Plasmodium Falciparum : Inhibition of Histone Acetylation and Generation of Reactive Oxygen Species. Antimicrob. Agents Chemother. 51, 488–494. 10.1128/aac.01238-06 17145789 PMC1797756

[B36] Cui L. Miao J. Furuya T. Fan Q. Li X. Rathod P. K. (2008b). Histone Acetyltransferase Inhibitor Anacardic Acid Causes Changes in Global Gene Expression during *In Vitro* Plasmodium Falciparum Development. Eukaryot. Cel 7, 1200–1210. 10.1128/ec.00063-08 PMC2446667 18487348

[B37] Cui L. Miao J. Furuya T. Li X. Su X.-z. Cui L. (2007b). PfGCN5-mediated Histone H3 Acetylation Plays a Key Role in Gene Expression in *Plasmodium Falciparum* . Eukaryot. Cel 6, 1219–1227. 10.1128/ec.00062-07 PMC1951105 17449656

[B38] Dastidar E. G. Dzeyk K. Krijgsveld J. Malmquist N. A. Doerig C. Scherf A. (2013). Comprehensive Histone Phosphorylation Analysis and Identification of Pf14-3-3 Protein as a Histone H3 Phosphorylation Reader in Malaria Parasites. PLoS One 8, e53179. 10.1371/journal.pone.0053179 23308157 PMC3538786

[B39] Delves M. J. (2012). *Plasmodium* Cell Biology Should Inform Strategies Used in the Development of Antimalarial Transmission-Blocking Drugs. Future Med. Chem. 4, 2251–2263. 10.4155/fmc.12.182 23234549

[B40] Dixon J. R. Selvaraj S. Yue F. Kim A. Li Y. Shen Y. (2012). Topological Domains in Mammalian Genomes Identified by Analysis of Chromatin Interactions. Nature 485, 376–380. 10.1038/nature11082 22495300 PMC3356448

[B41] Doug Chung D.-W. Ponts N. Cervantes S. Le Roch K. G. (2009). Post-translational Modifications in Plasmodium: More Than You Think! Mol. Biochem. Parasitol. 168, 123–134. 10.1016/j.molbiopara.2009.08.001 19666057

[B42] Dvorak J. A. Miller L. H. Whitehouse W. C. Shiroishi T. (1975). Invasion of Erythrocytes by Malaria Merozoites. Science 187, 748–750. 10.1126/science.803712 803712

[B43] Dyer M. Day K. (2000). Expression of *Plasmodium Falciparum* Trimeric G Proteins and Their Involvement in Switching to Sexual Development. Mol. Biochem. Parasitol. 110, 437–448. 10.1016/s0166-6851(00)00288-7 11071298

[B44] Fan Q. An L. Cui L. (2004a). PfADA2, a *Plasmodium Falciparum* Homologue of the Transcriptional Coactivator ADA2 and its *In Vivo* Association with the Histone Acetyltransferase PfGCN5. Gene 336, 251–261. 10.1016/j.gene.2004.04.005 15246536

[B45] Fan Q. An L. Cui L. (2004b). Plasmodium Falciparum Histone Acetyltransferase, a Yeast GCN5 Homologue Involved in Chromatin Remodeling. Eukaryot. Cel 3, 264–276. 10.1128/ec.3.2.264-276.2004 PMC387650 15075257

[B46] Fan Q. Miao J. Cui L. Cui L. (2009). Characterization of PRMT1 from Plasmodium Falciparum. Biochem. J. 421, 107–118. 10.1042/bj20090185 19344311 PMC8815339

[B48] Filarsky M. Fraschka S. A. Niederwieser I. Brancucci N. M. B. Carrington E. Carrió E. (2018). GDV1 Induces Sexual Commitment of Malaria Parasites by Antagonizing HP1-dependent Gene Silencing. Science 359, 1259–1263. 10.1126/science.aan6042 29590075 PMC6219702

[B49] Fischle W. Tseng B. S. Dormann H. L. Ueberheide B. M. Garcia B. A. Shabanowitz J. (2005). Regulation of HP1-Chromatin Binding by Histone H3 Methylation and Phosphorylation. Nature 438, 1116–1122. 10.1038/nature04219 16222246

[B50] Fleck K. Nitz M. Jeffers V. (2021). "Reading" a New Chapter in Protozoan Parasite Transcriptional Regulation. Plos Pathog. 17, e1010056. 10.1371/journal.ppat.1010056 34855919 PMC8638923

[B51] Flueck C. Bartfai R. Volz J. Niederwieser I. Salcedo-Amaya A. M. Alako B. T. F. (2009). *Plasmodium Falciparum* Heterochromatin Protein 1 marks Genomic Loci Linked to Phenotypic Variation of Exported Virulence Factors. Plos Pathog. 5, e1000569. 10.1371/journal.ppat.1000569 19730695 PMC2731224

[B52] Fraschka S. A.-K. Henderson R. W. M. Bártfai R. (2016). H3.3 Demarcates GC-Rich Coding and Subtelomeric Regions and Serves as Potential Memory Mark for Virulence Gene Expression in Plasmodium Falciparum. Sci. Rep. 6, 31965. 10.1038/srep31965 27555062 PMC4995406

[B53] Fraschka S. A. Filarsky M. Hoo R. Niederwieser I. Yam X. Y. Brancucci N. M. B. (2018). Comparative Heterochromatin Profiling Reveals Conserved and Unique Epigenome Signatures Linked to Adaptation and Development of Malaria Parasites. Cell Host & Microbe 23, 407–420. 10.1016/j.chom.2018.01.008 29503181 PMC5853956

[B54] Gardner M. J. Hall N. Fung E. White O. Berriman M. Hyman R. W. (2002). Genome Sequence of the Human Malaria Parasite *Plasmodium Falciparum* . Nature 419, 498–511. 10.1038/nature01097 12368864 PMC3836256

[B55] Goll M. G. Bestor T. H. (2002). Histone Modification and Replacement in Chromatin Activation: Figure 1. Genes Dev. 16, 1739–1742. 10.1101/gad.1013902 12130533

[B56] Gomes A. R. Bushell E. Schwach F. Girling G. Anar B. Quail M. A. (2015). A Genome-Scale Vector Resource Enables High-Throughput Reverse Genetic Screening in a Malaria Parasite. Cell Host & Microbe 17, 404–413. 10.1016/j.chom.2015.01.014 25732065 PMC4362957

[B57] Gómez-Díaz E. Yerbanga R. S. Lefèvre T. Cohuet A. Rowley M. J. Ouedraogo J. B. (2017). Epigenetic Regulation of Plasmodium Falciparum Clonally Variant Gene Expression during Development in Anopheles gambiae. Sci. Rep. 7, 40655. 10.1038/srep40655 28091569 PMC5238449

[B58] Govin J. Dorsey J. Gaucher J. Rousseaux S. Khochbin S. Berger S. L. (2010). Systematic Screen Reveals New Functional Dynamics of Histones H3 and H4 during Gametogenesis. Genes Dev. 24, 1772–1786. 10.1101/gad.1954910 20713519 PMC2922505

[B59] Goyal M. Heinberg A. Mitesser V. Kandelis-Shalev S. Singh B. K. Dzikowski R. (2021). Phosphorylation of the Canonical Histone H2A Marks Foci of Damaged DNA in Malaria Parasites. mSphere 6, e01131. 10.1128/mSphere.01131-20 33441412 PMC7845613

[B60] Green J. L. Wu Y. Encheva V. Lasonder E. Prommaban A. Kunzelmann S. (2020). Ubiquitin Activation Is Essential for Schizont Maturation in Plasmodium Falciparum Blood-Stage Development. Plos Pathog. 16, e1008640. 10.1371/journal.ppat.1008640 32569299 PMC7332102

[B61] Guillemette B. Drogaris P. Lin H.-H. S. Armstrong H. Hiragami-Hamada K. Imhof A. (2011). H3 Lysine 4 Is Acetylated at Active Gene Promoters and Is Regulated by H3 Lysine 4 Methylation. Plos Genet. 7, e1001354. 10.1371/journal.pgen.1001354 21483810 PMC3069113

[B62] Gupta A. P. Bozdech Z. (2017). Epigenetic Landscapes Underlining Global Patterns of Gene Expression in the Human Malaria Parasite, Plasmodium Falciparum. Int. J. Parasitol. 47, 399–407. 10.1016/j.ijpara.2016.10.008 28414071

[B63] Gupta A. P. Chin W. H. Zhu L. Mok S. Luah Y.-H. Lim E.-H. (2013). Dynamic Epigenetic Regulation of Gene Expression during the Life Cycle of Malaria Parasite *Plasmodium Falciparum* . Plos Pathog. 9, e1003170. 10.1371/journal.ppat.1003170 23468622 PMC3585154

[B64] Gupta A. P. Zhu L. Tripathi J. Kucharski M. Patra A. Bozdech Z. (2017). Histone 4 Lysine 8 Acetylation Regulates Proliferation and Host-Pathogen Interaction in *Plasmodium Falciparum* . Epigenetics & Chromatin 10, 40. 10.1186/s13072-017-0147-z 28830512 PMC5568195

[B65] Herrera-Solorio A. M. Vembar S. S. Macpherson C. R. Lozano-Amado D. Meza G. R. Xoconostle-Cazares B. (2019). Clipped Histone H3 Is Integrated into Nucleosomes of DNA Replication Genes in the Human Malaria Parasite Plasmodium Falciparum. EMBO Rep. 20, e46331. 10.15252/embr.201846331 30833341 PMC6446197

[B66] Hirota T. Lipp J. J. Toh B.-H. Peters J.-M. (2005). Histone H3 Serine 10 Phosphorylation by Aurora B Causes HP1 Dissociation from Heterochromatin. Nature 438, 1176–1180. 10.1038/nature04254 16222244

[B67] Hoeijmakers W. A. M. Miao J. Schmidt S. Toenhake C. G. Shrestha S. Venhuizen J. (2019). Epigenetic Reader Complexes of the Human Malaria Parasite, *Plasmodium Falciparum* . Nucleic Acids Res. 47, 11574–11588. 10.1093/nar/gkz1044 31728527 PMC7145593

[B68] Hoeijmakers W. A. M. Salcedo‐Amaya A. M. Smits A. H. Françoijs K. J. Treeck M. Gilberger T. W. (2013). H 2 A . Z/H 2 B . Z Double‐variant Nucleosomes Inhabit the at ‐rich Promoter Regions of the P Lasmodium Falciparum Genome. Mol. Microbiol. 87, 1061–1073. 10.1111/mmi.12151 23320541 PMC3594968

[B69] Hoeijmakers W. A. M. Stunnenberg H. G. Bártfai R. (2012). Placing the *Plasmodium Falciparum* Epigenome on the Map. Trends Parasitol. 28, 486–495. 10.1016/j.pt.2012.08.006 22999479

[B70] Hunter T. (2007). The Age of Crosstalk: Phosphorylation, Ubiquitination, and beyond. Mol. Cel 28, 730–738. 10.1016/j.molcel.2007.11.019 18082598

[B71] Issar N. Ralph S. A. Mancio-Silva L. Keeling C. Scherf A. (2009). Differential Sub-nuclear Localisation of Repressive and Activating Histone Methyl Modifications in P. Falciparum. Microbes Infect. 11, 403–407. 10.1016/j.micinf.2008.12.010 19136073

[B72] Issar N. Roux E. Mattei D. Scherf A. (2008). Identification of a Novel post-translational Modification inPlasmodium Falciparum: Protein Sumoylation in Different Cellular Compartments. Cell Microbiol 10, 1999–2011. 10.1111/j.1462-5822.2008.01183.x 18547337 PMC2613257

[B73] Janssen A. Colmenares S. U. Lee T. Karpen G. H. (2019). Timely Double-Strand Break Repair and Pathway Choice in Pericentromeric Heterochromatin Depend on the Histone Demethylase dKDM4A. Genes Dev. 33, 103–115. 10.1101/gad.317537.118 30578303 PMC6317320

[B74] Jiang L. Mu J. Zhang Q. Ni T. Srinivasan P. Rayavara K. (2013). PfSETvs Methylation of Histone H3K36 Represses Virulence Genes in *Plasmodium Falciparum* . Nature 499, 223–227. 10.1038/nature12361 23823717 PMC3770130

[B75] Joice R. Nilsson S. K. Montgomery J. Dankwa S. Egan E. Morahan B. (2014). *Plasmodium Falciparum* Transmission Stages Accumulate in the Human Bone Marrow. Sci. Transl Med. 6, 244re5. 10.1126/scitranslmed.3008882 PMC4175394 25009232

[B76] Josling G. A. Llinás M. (2015). Sexual Development in *Plasmodium* Parasites: Knowing when It's Time to Commit. Nat. Rev. Microbiol. 13, 573–587. 10.1038/nrmicro3519 26272409

[B77] Josling G. A. Russell T. J. Venezia J. Orchard L. Van Biljon R. Painter H. J. (2020). Dissecting the Role of PfAP2-G in Malaria Gametocytogenesis. Nat. Commun. 11, 1503. 10.1038/s41467-020-15026-0 32198457 PMC7083873

[B78] Jung H. R. Sidoli S. Haldbo S. Sprenger R. R. Schwämmle V. Pasini D. (2013). Precision Mapping of Coexisting Modifications in Histone H3 Tails from Embryonic Stem Cells by ETD-MS/MS. Anal. Chem. 85, 8232–8239. 10.1021/ac401299w 23889513

[B79] Kafsack B. F. C. Rovira-Graells N. Clark T. G. Bancells C. Crowley V. M. Campino S. G. (2014). A Transcriptional Switch Underlies Commitment to Sexual Development in Malaria Parasites. Nature 507, 248–252. 10.1038/nature12920 24572369 PMC4040541

[B80] Kanyal A. Rawat M. Gurung P. Choubey D. Anamika K. Karmodiya K. (2018). Genome‐wide Survey and Phylogenetic Analysis of Histone Acetyltransferases and Histone Deacetylases of Plasmodium Falciparum. FEBS J. 285, 1767–1782. 10.1111/febs.14376 29284196

[B81] Kappe S. H. I. Buscaglia C. A. Nussenzweig V. (2004). *Plasmodium* Sporozoite Molecular Cell Biology. Annu. Rev. Cel Dev. Biol. 20, 29–59. 10.1146/annurev.cellbio.20.011603.150935 15473834

[B82] Karmodiya K. Pradhan S. J. Joshi B. Jangid R. Reddy P. C. Galande S. (2015). A Comprehensive Epigenome Map of *Plasmodium Falciparum* Reveals Unique Mechanisms of Transcriptional Regulation and Identifies H3K36me2 as a Global Mark of Gene Suppression. Epigenetics & Chromatin 8, 32. 10.1186/s13072-015-0029-1 26388940 PMC4574195

[B83] Kaur I. Zeeshan M. Saini E. Kaushik A. Mohmmed A. Gupta D. (2016). Widespread Occurrence of Lysine Methylation in Plasmodium Falciparum Proteins at Asexual Blood Stages. Sci. Rep. 6, 35432. 10.1038/srep35432 27762281 PMC5071865

[B84] Keogh M.-C. Kurdistani S. K. Morris S. A. Ahn S. H. Podolny V. Collins S. R. (2005). Cotranscriptional Set2 Methylation of Histone H3 Lysine 36 Recruits a Repressive Rpd3 Complex. Cell 123, 593–605. 10.1016/j.cell.2005.10.025 16286008

[B85] Kumar M. Skillman K. Duraisingh M. T. (2021). Linking Nutrient Sensing and Gene Expression in Plasmodium Falciparum Blood‐stage Parasites. Mol. Microbiol. 115, 891–900. 10.1111/mmi.14652 33236377 PMC8144236

[B86] Lasonder E. Rijpma S. R. van Schaijk B. C. L. Hoeijmakers W. A. M. Kensche P. R. Gresnigt M. S. (2016). Integrated Transcriptomic and Proteomic Analyses ofP. Falciparumgametocytes: Molecular Insight into Sex-specific Processes and Translational Repression. Nucleic Acids Res. 44, 6087–6101. 10.1093/nar/gkw536 27298255 PMC5291273

[B87] Latchman D. S. (2010). “Role of Chromatin Structure in Gene Control,,” in Gene Control. Editor Owen E. (New York: Garland Science), 66–73.

[B88] Lee S.-B. Jasencakova Z. Groth A. (2012). H3K56me1 marks a Spot for PCNA. Mol. Cel 46, 1–2. 10.1016/j.molcel.2012.03.022 22500733

[B89] Lemieux J. E. Kyes S. A. Otto T. D. Feller A. I. Eastman R. T. Pinches R. A. (2013). Genome‐wide Profiling of Chromosome Interactions in P Lasmodium Falciparum Characterizes Nuclear Architecture and Reconfigurations Associated with Antigenic Variation. Mol. Microbiol. 90, 519–537. 10.1111/mmi.12381 23980881 PMC3894959

[B90] López-Barragán M. J. Lemieux J. Quiñones M. Williamson K. C. Molina-Cruz A. Cui K. (2011). Directional Gene Expression and Antisense Transcripts in Sexual and Asexual Stages of *Plasmodium Falciparum* . BMC Genomics 12, 587. 10.1186/1471-2164-12-587 22129310 PMC3266614

[B91] Lopez-Rubio J.-J. Mancio-Silva L. Scherf A. (2009). Genome-wide Analysis of Heterochromatin Associates Clonally Variant Gene Regulation with Perinuclear Repressive Centers in Malaria Parasites. Cell Host & Microbe 5, 179–190. 10.1016/j.chom.2008.12.012 19218088

[B92] Maltby V. E. Martin B. J. E. Schulze J. M. Johnson I. Hentrich T. Sharma A. (2012). Histone H3 Lysine 36 Methylation Targets the Isw1b Remodeling Complex to Chromatin. Mol. Cell Biol. 32, 3479–3485. 10.1128/mcb.00389-12 22751925 PMC3422011

[B93] Mancio-Silva L. Lopez-Rubio J. J. Claes A. Scherf A. (2013). Sir2a Regulates rDNA Transcription and Multiplication Rate in the Human Malaria Parasite Plasmodium Falciparum. Nat. Commun. 4, 1530. 10.1038/ncomms2539 23443558 PMC3586713

[B94] Matthews K. A. Senagbe K. M. Nötzel C. Gonzales C. A. Tong X. Rijo-Ferreira F. (2020). Disruption of the *Plasmodium Falciparum* Life Cycle through Transcriptional Reprogramming by Inhibitors of Jumonji Demethylases. ACS Infect. Dis. 6, 1058–1075. 10.1021/acsinfecdis.9b00455 32272012 PMC7748244

[B95] Merrick C. J. Duraisingh M. T. (2010). Epigenetics in Plasmodium: what Do We Really Know? Eukaryot. Cel 9, 1150–1158. 10.1128/ec.00093-10 PMC2918939 20562224

[B96] Meshorer E. Yellajoshula D. George E. Scambler P. J. Brown D. T. Misteli T. (2006). Hyperdynamic Plasticity of Chromatin Proteins in Pluripotent Embryonic Stem Cells. Develop. Cel 10, 105–116. 10.1016/j.devcel.2005.10.017 PMC1868458 16399082

[B97] Miao J. Fan Q. Cui L. Li J. Li J. Cui L. (2006). The Malaria Parasite *Plasmodium Falciparum* Histones: Organization, Expression, and Acetylation. Gene 369, 53–65. 10.1016/j.gene.2005.10.022 16410041

[B98] Miao J. Fan Q. Cui L. Li X. Wang H. Ning G. (2010a). The MYST Family Histone Acetyltransferase Regulates Gene Expression and Cell Cycle in Malaria Parasite *Plasmodium Falciparum* . Mol. Microbiol. 78, 883–902. 10.1111/j.1365-2958.2010.07371.x 20807207 PMC2978264

[B99] Miao J. Li J. Fan Q. Li X. Li X. Cui L. (2010b). The Puf-Family RNA-Binding Protein PfPuf2 Regulates Sexual Development and Sex Differentiation in the Malaria parasitePlasmodium Falciparum. J. Cel Sci 123, 1039–1049. 10.1242/jcs.059824 PMC2844316 20197405

[B100] Miao J. Wang C. Lucky A. B. Liang X. Min H. Adapa S. R. (2021). A Unique GCN5 Histone Acetyltransferase Complex Controls Erythrocyte Invasion and Virulence in the Malaria Parasite Plasmodium Falciparum. Plos Pathog. 17, e1009351. 10.1371/journal.ppat.1009351 34403450 PMC8396726

[B101] Miller J. L. Grant P. A. (2013). The Role of DNA Methylation and Histone Modifications in Transcriptional Regulation in Humans. Subcell Biochem. 61, 289–317. 10.1007/978-94-007-4525-4_13 23150256 PMC6611551

[B102] Müller M. M. Muir T. W. (2015). Histones: at the Crossroads of Peptide and Protein Chemistry. Chem. Rev. 115, 2296–2349. 10.1021/cr5003529 25330018 PMC4378460

[B103] Ngwa C. J. Gross M. R. Musabyimana J. P. Pradel G. Deitsch K. W. (2021). The Role of the Histone Methyltransferase PfSET10 in Antigenic Variation by Malaria Parasites: a Cautionary Tale. mSphere 6, e01217. 10.1128/mSphere.01217-20 33536326 PMC7860991

[B104] Ngwa C. J. Kiesow M. J. Papst O. Orchard L. M. Filarsky M. Rosinski A. N. (2017). Transcriptional Profiling Defines Histone Acetylation as a Regulator of Gene Expression during Human-To-Mosquito Transmission of the Malaria Parasite *Plasmodium Falciparum* . Front. Cel. Infect. Microbiol. 7, 320. 10.3389/fcimb.2017.00320 PMC5522858 28791254

[B105] Perez-Toledo K. Rojas-Meza A. P. Mancio-Silva L. Hernandez-Cuevas N. A. Delgadillo D. M. Vargas M. (2009). *Plasmodium Falciparum* Heterochromatin Protein 1 Binds to Tri-methylated Histone 3 Lysine 9 and Is Linked to Mutually Exclusive Expression of *Var* Genes. Nucleic Acids Res. 37, 2596–2606. 10.1093/nar/gkp115 19270070 PMC2677873

[B106] Petter M. Lee C. C. Byrne T. J. Boysen K. E. Volz J. Ralph S. A. (2011). Expression of *P. Falciparum Var* Genes Involves Exchange of the Histone Variant H2A.Z at the Promoter. Plos Pathog. 7, e1001292. 10.1371/journal.ppat.1001292 21379342 PMC3040674

[B107] Petter M. Selvarajah S. A. Lee C. C. Chin W. H. Gupta A. P. Bozdech Z. (2013). H2A.Z and H2B.Z Double-Variant Nucleosomes Define Intergenic Regions and Dynamically Occupyvargene Promoters in the Malaria parasitePlasmodium Falciparum. Mol. Microbiol. 87, 1167–1182. 10.1111/mmi.12154 23373537

[B108] Poran A. Nötzel C. Aly O. Mencia-Trinchant N. Harris C. T. Guzman M. L. (2017). Single-cell RNA Sequencing Reveals a Signature of Sexual Commitment in Malaria Parasites. Nature 551, 95–99. 10.1038/nature24280 29094698 PMC6055935

[B109] Prata I. O. Cubillos E. F. G. Krüger A. Barbosa D. Martins J. Jr. Setubal J. C. (2021). *Plasmodium Falciparum* Acetyl-CoA Synthetase Is Essential for Parasite Intraerythrocytic Development and Chromatin Modification. ACS Infect. Dis. 7, 3224–3240. 10.1021/acsinfecdis.1c00414 34766750

[B110] Read D. F. Cook K. Lu Y. Y. Le Roch K. G. Noble W. S. (2019). Predicting Gene Expression in the Human Malaria Parasite Plasmodium Falciparum Using Histone Modification, Nucleosome Positioning, and 3D Localization Features. Plos Comput. Biol. 15, e1007329. 10.1371/journal.pcbi.1007329 31509524 PMC6756558

[B111] Reader J. Van Der Watt M. E. Taylor D. Le Manach C. Mittal N. Ottilie S. (2021). Multistage and Transmission-Blocking Targeted Antimalarials Discovered from the Open-Source MMV Pandemic Response Box. Nat. Commun. 12, 269. 10.1038/s41467-020-20629-8 33431834 PMC7801607

[B112] Reiter K. H. Ramachandran A. Xia X. Boucher L. E. Bosch J. Matunis M. J. (2016). Characterization and Structural Insights into Selective E1-E2 Interactions in the Human and Plasmodium Falciparum SUMO Conjugation Systems. J. Biol. Chem. 291, 3860–3870. 10.1074/jbc.m115.680801 26697886 PMC4759166

[B113] Ren W. Fan H. Grimm S. A. Kim J. J. Li L. Guo Y. (2021). DNMT1 Reads Heterochromatic H4K20me3 to Reinforce LINE-1 DNA Methylation. Nat. Commun. 12, 2490. 10.1038/s41467-021-22665-4 33941775 PMC8093215

[B114] Rovira-Graells N. Gupta A. P. Planet E. Crowley V. M. Mok S. Ribas De Pouplana L. (2012). Transcriptional Variation in the Malaria Parasite Plasmodium Falciparum. Genome Res. 22, 925–938. 10.1101/gr.129692.111 22415456 PMC3337437

[B115] Ruthenburg A. J. Li H. Milne T. A. Dewell S. Mcginty R. K. Yuen M. (2011). Recognition of a Mononucleosomal Histone Modification Pattern by BPTF via Multivalent Interactions. Cell 145, 692–706. 10.1016/j.cell.2011.03.053 21596426 PMC3135172

[B116] Ryu H.-Y. Hochstrasser M. (2021). Histone Sumoylation and Chromatin Dynamics. Nucleic Acids Res. 49, 6043–6052. 10.1093/nar/gkab280 33885816 PMC8216275

[B117] Sabari B. R. Tang Z. Huang H. Yong-Gonzalez V. Molina H. Kong H. E. (2015). Intracellular Crotonyl-CoA Stimulates Transcription through P300-Catalyzed Histone Crotonylation. Mol. Cel 58, 203–215. 10.1016/j.molcel.2015.02.029 PMC4501262 25818647

[B118] Salcedo-Amaya A. M. Van Driel M. A. Alako B. T. Trelle M. B. Van Den Elzen A. M. G. Cohen A. M. (2009). Dynamic Histone H3 Epigenome Marking during the Intraerythrocytic Cycle of *Plasmodium Falciparum* . Proc. Natl. Acad. Sci. U.S.A. 106, 9655–9660. 10.1073/pnas.0902515106 19497874 PMC2701018

[B119] Sanderson T. Rayner J. C. (2017). PhenoPlasm: a Database of Disruption Phenotypes for Malaria Parasite Genes. Wellcome Open Res. 2, 45. 10.12688/wellcomeopenres.11896.1 28748223 PMC5500895

[B120] Santos-Rosa H. Millán-Zambrano G. Han N. Leonardi T. Klimontova M. Nasiscionyte S. (2021). Methylation of Histone H3 at Lysine 37 by Set1 and Set2 Prevents Spurious DNA Replication. Mol. Cel 81, 2793–2807. 10.1016/j.molcel.2021.04.021 PMC7612968 33979575

[B121] Saraf A. Cervantes S. Bunnik E. M. Ponts N. Sardiu M. E. Chung D.-W. D. (2016). Dynamic and Combinatorial Landscape of Histone Modifications during the Intraerythrocytic Developmental Cycle of the Malaria Parasite. J. Proteome Res. 15, 2787–2801. 10.1021/acs.jproteome.6b00366 27291344 PMC5905347

[B122] Shang X. Shen S. Tang J. He X. Zhao Y. Wang C. (2021). A cascade of Transcriptional Repression Determines Sexual Commitment and Development in *Plasmodium Falciparum* . Nucleic Acids Res. 49, 9264–9279. 10.1093/nar/gkab683 34365503 PMC8450074

[B123] Sherman I. W. (1979). Biochemistry of *Plasmodium* (Malarial Parasites). Microbiol. Rev. 43, 453–495. 10.1128/mr.43.4.453-495.1979 94424 PMC281489

[B124] Silberhorn E. Schwartz U. Löffler P. Schmitz S. Symelka A. De Koning-Ward T. (2016). Plasmodium Falciparum Nucleosomes Exhibit Reduced Stability and Lost Sequence Dependent Nucleosome Positioning. Plos Pathog. 12, e1006080. 10.1371/journal.ppat.1006080 28033404 PMC5198986

[B125] Sinden R. E. Canning E. U. Bray R. S. Smalley M. E. (1978). Gametocyte and Gamete Development in *Plasmodium Falciparum* . Proc. R. Soc. Lond. B Biol. Sci. 201, 375–399. 10.1098/rspb.1978.0051 27809

[B126] Singh S. Santos J. M. Orchard L. M. Yamada N. Van Biljon R. Painter H. J. (2020). The PfAP2-G2 Transcription Factor Is a Critical Regulator of Gametocyte Maturation. Mol. Microbiol. 115, 1005–1024. 10.1111/mmi.14676 PMC8330521 33368818

[B127] Sinha A. Hughes K. R. Modrzynska K. K. Otto T. D. Pfander C. Dickens N. J. (2014). A cascade of DNA-Binding Proteins for Sexual Commitment and Development in *Plasmodium* . Nature 507, 253–257. 10.1038/nature12970 24572359 PMC4105895

[B128] Smalley M. E. Abdalla S. Brown J. (1981). The Distribution of Plasmodium Falciparum in the Peripheral Blood and Bone Marrow of Gambian Children. Trans. R. Soc. Trop. Med. Hyg. 75, 103–105. 10.1016/0035-9203(81)90019-5 7022784

[B129] Sullivan W. J. Jr. Naguleswaran A. Angel S. O. (2006). Histones and Histone Modifications in Protozoan Parasites. Cel Microbiol 8, 1850–1861. 10.1111/j.1462-5822.2006.00818.x 17026479

[B130] Summers R. L. Pasaje C. F. A. Pisco J. P. Striepen J. Luth M. R. Kumpornsin K. (2021). Chemogenomics Identifies Acetyl-Coenzyme A Synthetase as a Target for Malaria Treatment and Prevention. Cell Chem Biol 29, 191. 10.1016/j.chembiol.2021.07.010 34348113 PMC8878317

[B131] Tan Y. Xue Y. Song C. Grunstein M. (2013). Acetylated Histone H3K56 Interacts with Oct4 to Promote Mouse Embryonic Stem Cell Pluripotency. Proc. Natl. Acad. Sci. U.S.A. 110, 11493–11498. 10.1073/pnas.1309914110 23798425 PMC3710873

[B132] Tang J. Chisholm S. A. Yeoh L. M. Gilson P. R. Papenfuss A. T. Day K. P. (2020). Histone Modifications Associated with Gene Expression and Genome Accessibility Are Dynamically Enriched at Plasmodium Falciparum Regulatory Sequences. Epigenetics & Chromatin 13, 50. 10.1186/s13072-020-00365-5 33225957 PMC7682024

[B133] Trager W. Gill G. S. (1992). Enhanced Gametocyte Formation in Young Erythrocytes byPlasmodium falciparumIn Vitro. J. Protozool 39, 429–432. 10.1111/j.1550-7408.1992.tb01476.x 1640389

[B134] Treeck M. Sanders J. L. Elias J. E. Boothroyd J. C. (2011). The Phosphoproteomes of *Plasmodium Falciparum* and *Toxoplasma Gondii* Reveal Unusual Adaptations within and beyond the Parasites' Boundaries. Cell Host & Microbe 10, 410–419. 10.1016/j.chom.2011.09.004 22018241 PMC3254672

[B135] Trelle M. B. Salcedo-Amaya A. M. Cohen A. M. Stunnenberg H. G. Jensen O. N. (2009). Global Histone Analysis by Mass Spectrometry Reveals a High Content of Acetylated Lysine Residues in the Malaria Parasite *Plasmodium Falciparum* . J. Proteome Res. 8, 3439–3450. 10.1021/pr9000898 19351122

[B136] Ukaegbu U. E. Kishore S. P. Kwiatkowski D. L. Pandarinath C. Dahan-Pasternak N. Dzikowski R. (2014). Recruitment of PfSET2 by RNA Polymerase II to Variant Antigen Encoding Loci Contributes to Antigenic Variation in *P. Falciparum* . Plos Pathog. 10, e1003854. 10.1371/journal.ppat.1003854 24391504 PMC3879369

[B137] Van Biljon R. Van Wyk R. Painter H. J. Orchard L. Reader J. Niemand J. (2019). Hierarchical Transcriptional Control Regulates *Plasmodium Falciparum* Sexual Differentiation. BMC Genomics 20, 920. 10.1186/s12864-019-6322-9 31795940 PMC6889441

[B138] Volz J. Carvalho T. G. Ralph S. A. Gilson P. Thompson J. Tonkin C. J. (2010). Potential Epigenetic Regulatory Proteins Localise to Distinct Nuclear Sub-compartments in *Plasmodium Falciparum* . Int. J. Parasitol. 40, 109–121. 10.1016/j.ijpara.2009.09.002 19765590

[B139] Volz J. C. Bártfai R. Petter M. Langer C. Josling G. A. Tsuboi T. (2012). PfSET10, a *Plasmodium Falciparum* Methyltransferase, Maintains the Active Var Gene in a Poised State during Parasite Division. Cell Host & Microbe 11, 7–18. 10.1016/j.chom.2011.11.011 22264509

[B140] Von Grüning H. Coradin M. Mendoza M. R. Reader J. Sidoli S. Garcia B. A. (2022). A Dynamic and Combinatorial Histone Code Drives Malaria Parasite Asexual and Sexual Development. Mol. Cell Proteomics, 100199. 10.1016/j.mcpro.2022.100199 35051657 PMC8941266

[B141] Walzer K. A. Kubicki D. M. Tang X. Chi J. T. (2018). Single-Cell Analysis Reveals Distinct Gene Expression and Heterogeneity in Male and Female *Plasmodium Falciparum* Gametocytes. mSphere 3, e00130. 10.1128/mSphere.00130-18 29643077 PMC5909122

[B142] Wang W.-F. Zhang Y.-L. (2020). PfSWIB, a Potential Chromatin Regulator for Var Gene Regulation and Parasite Development in Plasmodium Falciparum. Parasites Vectors 13, 48. 10.1186/s13071-020-3918-5 32019597 PMC7001229

[B143] Watzlowik M. T. Das S. Meissner M. Längst G. (2021). Peculiarities of Plasmodium Falciparum Gene Regulation and Chromatin Structure. Int. J. Mol. Sci. 22, 5168. 10.3390/ijms22105168 34068393 PMC8153576

[B144] WHO (2020). World Malaria Report 2020: 20 Years of Global Progress and Challenges. Geneva: World Health Organization.

[B145] Williams J. L. (1999). Stimulation of *Plasmodium Falciparum* Gametocytogenesis by Conditioned Medium from Parasite Cultures. Am. J. Trop. Med. Hyg. 60, 7–13. 10.4269/ajtmh.1999.60.7 9988315

[B146] Witmer K. Fraschka S. A. Vlachou D. Bártfai R. Christophides G. K. (2020). An Epigenetic Map of Malaria Parasite Development from Host to Vector. Sci. Rep. 10, 6354. 10.1038/s41598-020-63121-5 32286373 PMC7156373

[B147] Young J. A. Fivelman Q. L. Blair P. L. De La Vega P. Le Roch K. G. Zhou Y. (2005). The *Plasmodium Falciparum* Sexual Development Transcriptome: a Microarray Analysis Using Ontology-Based Pattern Identification. Mol. Biochem. Parasitol. 143, 67–79. 10.1016/j.molbiopara.2005.05.007 16005087

[B148] Yu Y. Song C. Zhang Q. Dimaggio P. A. Garcia B. A. York A. (2012). Histone H3 Lysine 56 Methylation Regulates DNA Replication through its Interaction with PCNA. Mol. Cel 46, 7–17. 10.1016/j.molcel.2012.01.019 PMC3327800 22387026

[B149] Zhang M. Wang C. Otto T. D. Oberstaller J. Liao X. Adapa S. R. (2018). Uncovering the Essential Genes of the Human Malaria Parasite *Plasmodium Falciparum* by Saturation Mutagenesis. Science 360, eaap7847. 10.1126/science.aap7847 29724925 PMC6360947

[B150] Zhao Y. Garcia B. A. (2015). Comprehensive Catalog of Currently Documented Histone Modifications. Cold Spring Harb Perspect. Biol. 7, a025064. 10.1101/cshperspect.a025064 26330523 PMC4563710

[B151] Zheng H. Xie W. (2019). The Role of 3D Genome Organization in Development and Cell Differentiation. Nat. Rev. Mol. Cel Biol 20, 535–550. 10.1038/s41580-019-0132-4 31197269

